# Hospital volume–outcome relationship in total knee arthroplasty: a systematic review and dose–response meta-analysis

**DOI:** 10.1007/s00167-021-06692-8

**Published:** 2021-09-08

**Authors:** C. M. Kugler, K. Goossen, T. Rombey, K. K. De Santis, T. Mathes, J. Breuing, S. Hess, R. Burchard, D. Pieper

**Affiliations:** 1 Institute for Research in Operative Medicine (IFOM), Witten/Herdecke University, Ostmerheimer Str. 200, 51109 Cologne, Germany; 2 Department of Health Care Management, Technische Universität Berlin, Straße des 17. Juni 135, 10623 Berlin, Germany; 3 Leibniz Institute for Prevention Research and Epidemiology (BIPS), Achterstr. 30, 28359 Bremen, Germany; 4 Department of Orthopaedics and Trauma Surgery, Lahn-Dill-Kliniken, Rotebergstr. 2, 35683 Dillenburg, Germany; 5 Department of Health, Witten/Herdecke University, Alfred-Herrhausen-Straße 50, 58448 Witten, Germany; 6 Department of Orthopaedics and Traumatology, University of Giessen and Marburg, Baldingerstraße, 35032 Marburg, Germany

**Keywords:** Total knee arthroplasty (TKA), Knee osteoarthritis, Hospital volume, Hospital volume–outcome relationship, Systematic review, Dose–response meta-analysis

## Abstract

**Purpose:**

This systematic review and dose–response meta-analysis aimed to investigate the relationship between hospital volume and outcomes for total knee arthroplasty (TKA).

**Methods:**

MEDLINE, Embase, CENTRAL and CINAHL were searched up to February 2020 for randomised controlled trials and cohort studies that reported TKA performed in hospitals with at least two different volumes and any associated patient-relevant outcomes. The adjusted effect estimates (odds ratios, OR) were pooled using a random-effects, linear dose–response meta-analysis. Heterogeneity was quantified using the *I*^2^-statistic. ROBINS-I and the GRADE approach were used to assess the risk of bias and the confidence in the cumulative evidence, respectively.

**Results:**

A total of 68 cohort studies with data from 1985 to 2018 were included. The risk of bias for all outcomes ranged from moderate to critical. Higher hospital volume may be associated with a lower rate of early revision ≤ 12 months (narrative synthesis of *k* = 7 studies, *n* = 301,378 patients) and is likely associated with lower mortality ≤ 3 months (OR = 0.91 per additional 50 TKAs/year, 95% confidence interval [0.87–0.95], *k* = 9, *n* = 2,638,996, *I*^2^ = 51%) and readmissions ≤ 3 months (OR = 0.98 [0.97–0.99], *k* = 3, *n* = 830,381, *I*^2^ = 44%). Hospital volume may not be associated with the rates of deep infections within 1–4 years, late revision (1–10 years) or adverse events ≤ 3 months. The confidence in the cumulative evidence was moderate for mortality and readmission rates; low for early revision rates; and very low for deep infection, late revision and adverse event rates.

**Conclusion:**

An inverse volume–outcome relationship probably exists for some TKA outcomes, including mortality and readmissions, and may exist for early revisions. Small reductions in unfavourable outcomes may be clinically relevant at the population level, supporting centralisation of TKA to high-volume hospitals.

**Level of evidence:**

III.

**Registration number:**

The study was registered in the International Prospective Register of Systematic Reviews (PROSPERO CRD42019131209 available at: https://www.crd.york.ac.uk/prospero/display_record.php?RecordID=131209).

**Supplementary Information:**

The online version contains supplementary material available at 10.1007/s00167-021-06692-8.

## Introduction

Total knee arthroplasty (TKA) can improve pain and function in patients with end-stage knee osteoarthritis [99] and is increasingly performed worldwide [48, 87]. Unfavourable outcomes of TKA include revision surgery, deep infection, readmissions, and mortality, though rates of mortality are low [12, 24, 87].

A hospital volume–outcome relationship exists for various surgical procedures, meaning that higher hospital volume is associated with improved health outcomes [59, 84]. Some countries have therefore centralised selected surgical procedures to high-volume hospitals [70, 86]. A volume–outcome relationship may also exist for TKA [36, 84, 106]. Previous systematic reviews [26, 62, 107] are likely out of date, and have methodical limitations. The only published meta-analysis compared TKA outcomes only between the highest and lowest hospital volume categories [107].

The aim of this systematic review was to quantify the relationship between hospital volume and patient-relevant outcomes of TKA including complications using a dose–response meta-analysis. The hypothesis was that, as with other surgical procedures, a higher hospital volume would be associated with better patient-relevant outcomes of TKA.

## Methods

The reporting of this systematic review adheres to the Preferred Reporting Items for Systematic Reviews and Meta-Analyses (PRISMA) 2020 Statement [80]. The protocol was registered prospectively in the Prospective Register of Systematic Reviews (PROSPERO registration number CRD42019131209 [89] and published upfront [90]. 

### Systematic literature search

The search strategies were developed with the support of an experienced librarian according to the Peer Review of Electronic Search Strategies (PRESS) guideline [63]. The electronic search was conducted without any limits in four databases (MEDLINE, Embase, CENTRAL, CINAHL; Supplementary Material 1) from inception to February 2020 and in trial registers (ClinicalTrials.gov, German Clinical Study Register, International Clinical Trials Registry Platform). Further sources of literature included conference proceedings, reference lists of included studies, forward citation searching (Web of Science) and contact with experts (Supplementary Table 1). No language restriction was applied. Articles published in languages other than English, German, or Italian were sent for professional translation.

### Study selection

Studies with any design that (1) involved patients undergoing primary and/or revision TKA, (2) reported data for at least two different hospital volumes, and (3) analysed at least one patient-relevant outcome were included (see Supplementary Table 2 for a full list of eligibility criteria). After the duplicates were removed, two reviewers independently screened the titles and abstracts of all retrieved sources in EndNote (Clarivate Analytics, version X9.1) and assessed the full text of all potentially eligible articles. Any discrepancies were resolved by consensus or, when necessary, by consultation with a third reviewer.

### Data extraction

Data were extracted independently by two reviewers using standardised data extraction sheets. Any discrepancies were resolved by consensus. The data items included study, patient, hospital and surgeon characteristics; time and country of data collection; data source; hospital volume definitions; TKA details; patient-relevant outcomes; and statistical analysis details (effect size types, confidence intervals, and confounding factors). The primary outcome was the early revision rate ≤ 12 months after TKA. The secondary outcomes were any other patient-relevant outcomes that were classified according to clinical experience as ‘main outcomes’ [41] or ‘other outcomes’. All extracted outcomes are summarised and defined in Supplementary Table 3. Study results (adjusted and/or unadjusted) were extracted separately for each hospital volume category and outcome. If data were missing or incompletely reported, study authors were contacted via email [37].

### Risk of bias and publication bias

The risk of bias in the included studies was independently assessed at the outcome level by two reviewers using the Risk Of Bias In Non-randomised Studies of Interventions (ROBINS-I) tool [108]. For any outcomes with at least ten studies, assessment of publication bias was planned by visual inspection of the funnel plots for asymmetry and by applying Egger’s [31] and Begg’s tests [10].

### Statistical analysis

Hospital volume was defined as the mean annual number of patients undergoing TKA. Hospital volume categories were standardised using their midpoints. For individual study outcomes, odds ratios (ORs) with 95% confidence intervals (95% CIs) were converted such that the lowest volume category was the reference.

Individual study results were plotted to visually inspect linearity (e.g. better outcomes with increasing volume) for each outcome. A random-effects linear dose–response meta-analysis according to Greenland and Longnecker [38] was used to pool ORs for outcomes reported in at least three studies with sufficient data (Supplementary Material 2). For each outcome, measurements ≤ 3 months after TKA were aggregated in one analysis and those > 3 months in another. Revisions were aggregated in three analyses: ≤ 12 months, 1–5 years, and 6–10 years after TKA. Wherever the overlap among two or more study samples exceeded 20%, only one study was selected for meta-analysis based on data completeness, sample size, and the suitability of the volume categories as criteria (Supplementary Tables 4, 5, 6)*.* The main dose–response meta-analysis was computed using the ‘best-adjusted’ effect estimates. These were the ORs adjusted for at least one confounding variable, including age, gender, and comorbidities, but not for post- or within-intervention variables such as surgeon volume. Heterogeneity between studies was assessed using the *Q* test and *I*^2^-statistic [46]. Four sensitivity analyses (Supplementary Material 3) were conducted; the first analysis compared extreme volume categories (highest vs. lowest), and the second, third and fourth analyses (post hoc) studied the influence of confounding variables. An additional post hoc dose–response meta-analysis was conducted using ‘best available’ (adjusted and unadjusted) effect estimates. All meta-analyses were performed with R 3.6.3 (R Foundation for Statistical Computing, Vienna, Austria) using the metafor and dosresmeta packages [25, 116]. Outcomes that were not suitable for meta-analysis (Supplementary Material 2) were synthesised narratively using the Synthesis Without Meta-analysis (SWiM) guideline (Supplementary Material 4) [20].

### Grading the evidence

Confidence in the cumulative evidence was evaluated using the Grading of Recommendations, Assessment, Development, and Evaluation (GRADE) approach [19, 41, 91, 95, 113] and applying Murad’s approach [72] for SWiM outcomes. Two reviewers independently graded outcomes using GRADEpro GDT software [64] and reached consensus during discussion.

### Patient involvement

Potential TKA patients were asked for their opinions on the hospital volume–outcome relationship for TKA and their hospital preferences using qualitative methodology (focus groups and interviews). The methods and results are reported elsewhere [55].

## Results

### Study identification and selection

**Fig. 1:**
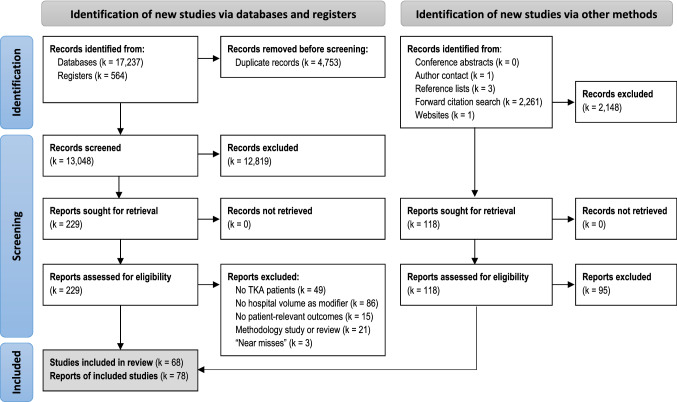
PRISMA flow diagram showing selection of articles for review

A total of 13,048 records were identified from electronic databases and trial registers, and 2266 were identified from reference lists of included articles, forward citation search, websites, and author contact. Of 347 full-text reports, 269 were excluded (Supplementary Table 7). This review included 68 cohort studies reported in 78 articles [1–9, 13, 16–18, 21–24, 27–30, 32–35, 39, 40, 42–45, 47, 49–54, 57, 58, 60, 61, 65, 67, 68, 71, 73–79, 81–83, 85, 88, 92–94, 97, 98, 100–104, 110–112, 114, 115, 118–122] with data representing the years from 1985 to 2018 (Fig. 1).

### Study and patient characteristics

The majority of studies used data from North America, while 22 used data from Europe, 9 from Asia and 1 from Australia. The data were obtained from administrative databases in 47 studies, clinical registries in 18 studies, and questionnaires in three studies. The average number of patients across all studies was 222,038 (data from 65 studies), with a median of 65% females (IQR 62–69%, data from 56 studies). The patients had a weighted mean age of 71 years (data from 40 studies). Each study included a median of 486 hospitals (IQR: 43–569, data from 51 studies). In 55 studies, the population was limited to primary TKA patients, 12 included primary and revision TKA patients, and one study did not specify the type of TKA. The study and patient characteristics of studies reporting primary and main secondary outcomes are shown in Table 1, and the characteristics of all 68 included studies are shown in Supplementary Table 8.

**Table 1:** Study characteristics with primary and main secondary outcomes

Study (references)	Study characteristics					Patients’ characteristics			Volume categories (per year)		Results	
	Type of funding	Country (region)	Primary data source	Data coll. (years)	No. of hospitals	No. of patients	% Female	Age (years)	Type	Upper limits; lower limit of highest category	Patient-relevant study outcomes	Authors’ conclusions favour
Anis 2019 [4]	n.r.	USA (OH, FL)	Clinical	2014–2017	16	12,541	62%	Mean ± SD: 69 ± 10	Thresholds	249; 500; ≥ 501	Infection	No evidence for a difference
Arias-de la Torre 2019 [5]	Non-profit	Spain (Catalonia)	Clinical	2005–2016	49	36,316	72%	≥ 65: 83%	Thresholds	124; ≥ 125	Early revision, mortality, revision	Lower volume
Arroyo 2018 [7]	None	USA (CA, FL, NY, MD)	Admin	2007–2014	752	739,857	63%	Mean ± SD: 67 ± 10	Hospital quartiles	145; 267; 487; ≥ 488	Readmission, infection	Higher volume
Badawy 2013 [9]	None	Norway	Clinical	1994–2010	54	26,698	68%	Mean: 71	Thresholds	24; 49; 99; 149; ≥ 150	Revision	Higher volume
Badawy 2017 [8]	None	Norway	Clinical	2005–2015	*67*	28,262	64%	Median (range): 70 (22–101)	Thresholds	49; 99; 149; ≥ 150	Infection	n.r.
Dy 2014 [30]	Non-profit	USA (CA, NY)	Admin	1997–2005	n.r.	301,955	36%	Median (IQR): 69 (61–76)	Thresholds	199; 400; ≥ 401	Revision	n.r.
Hervey 2003 [45]	Mixed^†^	USA	Admin	1997	n.r.	55,510	n.r.	n.r.	Thresholds	84; 149; 249; ≥ 250	Mortality, infection, AE, LOS	Higher volume
Jeschke 2017 [50]	n.r.	Germany	Admin	2012	966	45,165	68%	≥ 70: 59%	Hospital quintiles	56; 93; 144; 251; ≥ 252	Early revision, revision	Higher volume
Judge 2006 [51]	Non-profit	UK (England)	Admin	1997–2002	Unknown	*205,321*	*59%*	n.r.	Thresholds	50; 100; 250; 500; ≥ 501	Mortality, revision, readmission, LOS	Higher volume
Katz 2004 [52]	Non-profit	USA	Admin	2000	3122	80,904	67%	> 75: 41%	Thresholds	25; 100; 200; ≥ 201	Mortality, infection, AE	Higher volume
Kreder 2003 [54]	n.r.	Canada (ON)	Admin	1992–1996	88	14,352	62%	Mean: 70	Hospital quintiles*	47; 113; ≥ 114	Early revision, mortality, revision, infection, AE, LOS	Higher volume
Maman 2019 [60]	Non-profit	USA (NY, FL, MD, KY)	Admin	2007–2014	*827*	922,819	63%	Mean ± SD: 67 ± 10	Patient quartiles	*145; 267; 487;* ≥ *488*	Mortality, AE, LOS	Higher volume
Manley 2009 [61]	For-profit^$^	USA	Admin	1997–2004	n.r.	53,971	n.r.	n.r.	Thresholds	25; 100; 200; ≥ 201	Revision	Higher volume
Meehan 2014 [65]	None	USA (CA)	Admin	2005–2009	300	120,538	62%	≥ 65: 62%	Thresholds	49; 100; 200; ≥ 201	Early revision, infection	Higher volume
Namba 2013a [73]	n.r.	USA (CA, CO, GA, HI, NWR, MAR)	Clinical	2001–2010	48	64,017	63%	Mean ± SD: 67 ± 10	Thresholds	99; 199; ≥ 200	Revision	No evidence for a difference
Namba 2013b [74]	None	USA (6 regions)	Clinical	2001–2009	45	56,216	63%	Mean ± SD: 67 ± 10	Thresholds	99; 199; ≥ 200	Infection	Lower volume
Nimptsch 2017 [76]	n.r.	Germany	Admin	2006–2013	1 011	1,093,296	66%	n.r.	Thresholds	49; ≥ 50	Mortality, LOS	Higher volume
Norton 1998 [77]	Non-profit	USA	Admin	1985–1990	n.r.	295,473	n.r.	Mean: 74	Thresholds	20; 40; 80; ≥ 81	AE	Higher volume
Pamilo 2015 [81]	n.r.	Finland	Clinical	1998–2010	80	59,696	69%	≥ 70: 55%	Thresholds	99; 249; 449; ≥ 450	Revision, readmission, LOS	Results are inconsistent
Pamilo 2018 [82]	None	Finland	Clinical	2009–2013	4	*4256*	*65%*	Mean: *69*	Individual hospitals	184; 219; 251; 321	Early revision, mortality, LOS	n.r.
Paterson 2010 [83]	Non-profit	Canada (ON)	Admin	2000–2004	65	27,217	62%	≥ 70: 51%	Patient quartiles	130; 180; 270; ≥ 271	Early revision, mortality, surgical compl., LOS	Results are inconsistent
Schulze Raestrup 2006 [94]	n.r.	Germany (NRW)	Admin	2002–2003	218	31,657	n.r.	n.r.	Thresholds	49; 99; 199; 299; ≥ 300	Infection, wound compl., AE	Higher volume
Shin 2015 [97]	n.r.	Korea	Admin	2007–2012	n.r	260,068	88%	Mean ± SD: 69 ± 7	Thresholds	19; 199; ≥ 200	Revision	Higher volume
Singh 2011 [98]	Non-profit	USA (PA)	Admin	2001–2002	169	19,418	65%	Mean (IQR): 69 (60–75)	Thresholds	25; 100; 200; ≥ 201	Mortality, infection, AE	Higher volume
Soohoo 2006 [102]	None	USA (CA)	Admin	1991–2001	413	222,684	62%	Mean ± SD: 69 ± 10	Hospital quintiles*	Means: 13; 50; 145	Mortality, readmission, infection, AE	Higher volume
Wei 2010 [118]	None	Taiwan	Admin	2000–2003	295	31,618	74%	Mean: 74	Hospital quartiles*	6; 23; ≥ 24	Infection, AE, LOS	n.r.
Yu 2019 [122]	Non-profit	Taiwan	Admin	2007–2008	437	30,828	75%	Mean ± SD: 70 ± 8	Thresholds	74; ≥ 75	Readmission	No evidence for a difference

All studies were cohort studies. Unpublished data provided by study authors in italic

*admin*. administrative, *AE* postoperative adverse events, *CA* California, *CO* Colorado, *coll*. collection, *compl*. complications, *FL* Florida, *GA* Georgia, *HCUP* Health Care Utilization Project, *HI* Hawaii, *IL* Illinois, *IN* Indiana, *KY* Kentucky, *LOS* length of stay, *MAR* Mid-Atlantic region, *MD* Maryland, *MI* Michigan, *n.r.* not reported, *NC* North Carolina, *NRW* North-Rhine Westphalia, *NWR* North-West region, *NY* New York State, *OH* Ohio, *ON* Ontario, *PA* Pennsylvania, *QoL* quality of life, *SN* Saxony, *TN* Tennessee, *UK* United Kingdom, *USA* United States of America, *WA* Western Australia

^§^Includes funding by Zimmer, Smith & Nephew (medical devices co.)

^†^Includes funding by Bristol–Meyers Squibb (pharmaceutical co.)

^$^Stryker Orthopaedics, Inc. (medical devices co.)

^‡^Number of TKAs (number of patients not reported)

*Some quantiles were combined

### Study results

Individual study results are reported for all adjusted or unadjusted outcomes by hospital volume category in Supplementary Tables 4 and 9, respectively, and are summarised for the primary outcome (early revision rates) in Table 2.

**Table 2:** Study results and risk of bias for early revision

Study (references)	Study characteristics			Results		Risk of bias (ROBINS-I)
	Country	Time period (years)	No. of patients	Volume categories (TKA/year)	Effect measure	
Meehan 2014 [65]	USA	2005–2009	120,538	1–49 50–100 101–200 > 200	Crude rate 2.52% 2.32% 1.96% 1.78%	Serious
Pamilo 2018 [82]	Finland	1998–2010	59,696	No differences in revision rates between hospital volume with data from only four hospitals with similar TKA volumes		Serious
Manley 2009 [61]	USA	1997–2004	53,971	1–25 26–100 101–200 > 200	Adjusted OR [CI] 1.91 [0.76–4.83] 1.38 [0.84–2.26] 1.17 [0.74–1.87] 1.00	Serious
Jeschke 2017 [50]	Germany	2012	45,165	10–56 57–93 94–144 145–251 252–1648	Crude rate 5.19% 4.26% 3.81% 3.49% 3.34%	Serious
Arias-de la Torre 2019 [5]	Spain	2005–2016	36,316	< 125 ≥ 125	Crude rate; Kaplan–Meier rate [CI] 0.67%; 0.64% [0.53–0.77%] 1.24%; 1.15% [1.00–1.32%]	Moderate
Paterson 2010 [83]	Canada	2000–2004	27,217	10–130 131–180 181–270 > 270	Adjusted OR [CI] 1.00 0.64 [0.39–1.04] 0.62 [0.42–0.91] 0.50 [0.34–0.72]	Serious
Kreder 2003 [54]	Canada	1992–1996	14,352	< 48 48–113 > 113	Adjusted OR [CI] 2.23 [1.10–4.50] 1.57 [0.90–2.90] 1.00	Serious

*CI* confidence interval, *OR* odds ratio, *ROBINS-I* risk of bias in non-randomised studies of interventions tool, *TKA* total knee arthroplasty

### Risk of bias

The risk of bias was moderate for 30 study outcomes, serious for 168, and critical for 3 (Supplementary Table 10). Bias was suspected mostly due to potential confounding, since most effect estimates were not appropriately adjusted for age, gender, and comorbidity.

### Primary outcome: early revision rate

A higher hospital volume may be associated with a lower early revision rate (7 studies [5, 50, 54, 61, 65, 82, 83], narrative synthesis Table 2, low certainty evidence). Five studies with a high risk of bias, which accounted for 261,243 of 301,378 (87%) patients in total for this outcome [50, 54, 61, 65, 83], reported lower revision rates for higher volumes. In contrast, the only study with a moderate risk of bias [5] found that a higher hospital volume (> 125 TKAs/year) was associated with a higher early revision rate.

### Main secondary outcomes

The results of the linear dose–response meta-analysis of best-adjusted effect estimates are presented in Table 3 (main secondary outcomes), Supplementary Table 11 (other secondary outcomes) and Supplementary Table 12 (post hoc linear dose–response meta-analysis using ‘best available’ effect estimates).

**Table 3:** Results of linear dose–response meta-analysis of best-adjusted effect estimates (main secondary outcomes)

Outcome	*k*	(*n/N*) [%]	*I*^2^	Pooled *OR* [95% CI] for 50 TKA/year increase	Risk of bias (ROBINS-I)	References
Mortality (≤ 3 months)	9	4769/2,638,996 (0.2%)	51%	**0.91** [0.87–0.95]	Moderate^a^	[45, 51, 52, 54, 60, 76, 83, 98, 104]
Infection (deep) (1–4 years)	3	797/97,019 (0.8%)	0%	1.03 [0.97–1.09]	Serious^b^	[4, 8, 74]
Revision (1–5 years)	5	5498/163,520 (3.4%)	98%	0.96 [0.86–1.07]	Serious^c^	[5, 50, 51, 54, 73]
Readmission (≤ 3 months)	3	78,895/830,381 (9.5%)	44%	**0.98** [0.97–0.99]	Serious^c^	[7, 81, 122]

Statistically significant results in bold

*CI* confidence interval, *I*^*2*^ index for residual heterogeneity, *k* number of studies, *n* patients with event, *N* number of patients at risk, *OR* odds ratio, ROBINS-I risk of bias in non-randomised studies of interventions tool, *TKA* total knee arthroplasty

^a^Overall risk of bias was serious in five studies and moderate in four studies. Since studies with moderate risk of bias dominated the results (accounted for more than 80% of patients and events), we assume that the overall result is not seriously biased

^b^Overall risk of bias was serious in all studies

^c^Overall risk of bias was serious in all but one study, and moderate in one study

#### Revision

There was no evidence for a linear dose–response relationship between hospital volume and revision rate within 1–5 years (OR = 0.96 per 50 TKAs/year increase, 95% CI [0.86–1.07]; 5 studies [5, 50, 51, 54, 73], *I*^2^ = 98%, very low certainty, Table 3). This finding was robust to sensitivity analyses (Supplementary Tables 13, 14, 16).

The relationship between hospital volume and revision rate within 6–10 years was inconsistent (narrative synthesis, 5 studies [5, 9, 30, 81, 97], very low certainty).

#### Mortality

**Fig. 2:**
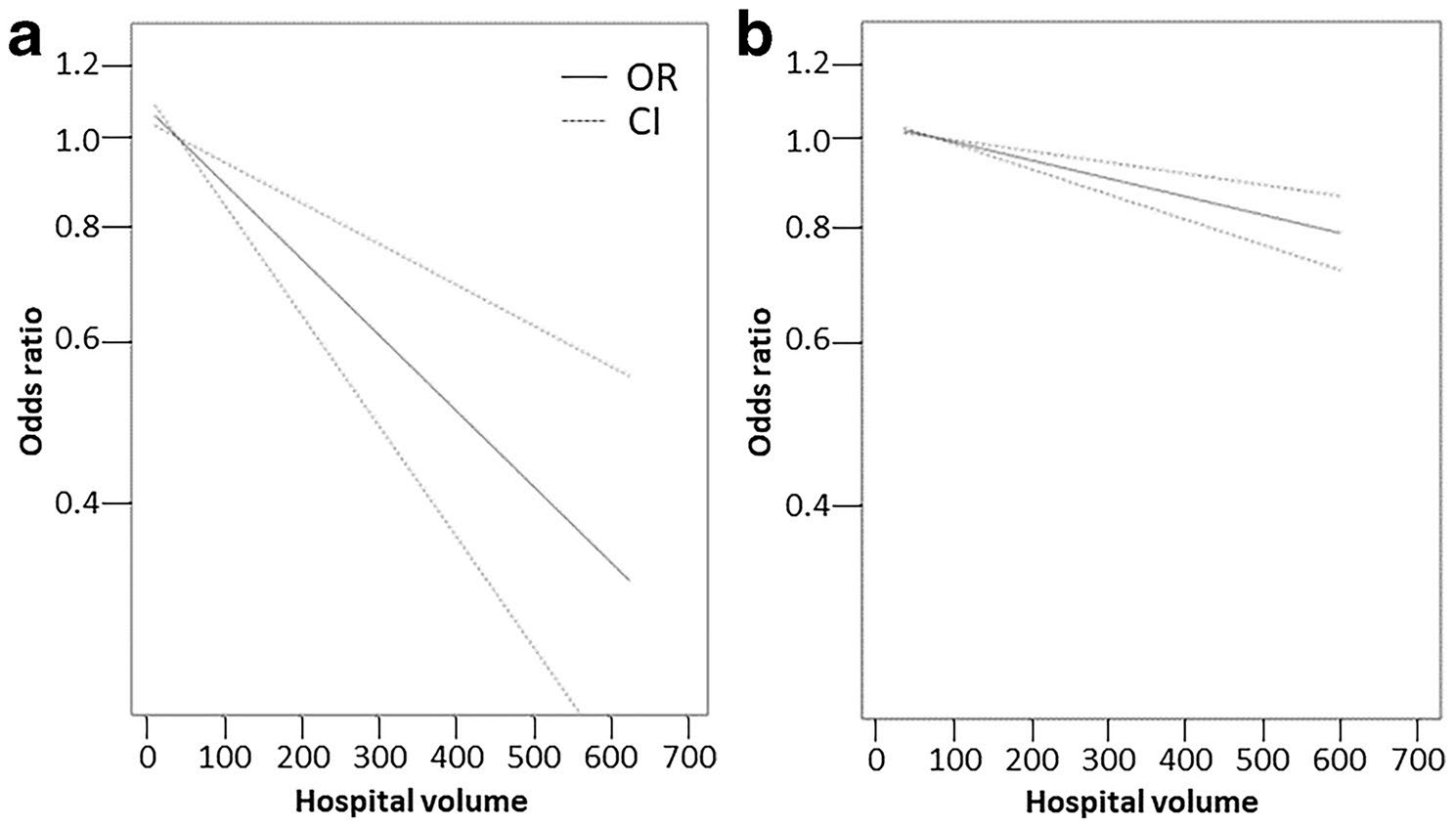
Linear dose–response meta-analysis for mortality (**a**) and readmission (**b**)

A higher hospital volume is likely associated with a lower mortality rate ≤ 3 months (OR = 0.91 per additional 50 TKAs/year, 95% CI [0.87–0.95]; 9 studies [45, 51, 52, 54, 60, 76, 83, 98, 104], *I*^2^ = 51%, moderate certainty, Table 3, Fig. 2a). The direction of this relationship was robust to sensitivity analyses (Supplementary Tables 13–16), although the pooled OR was no longer significant when the analysis included only data that were also adjusted for surgeon volume (Supplementary Table 15).

#### Deep infection

There was no evidence for a linear dose–response association between hospital volume and the rate of deep infection within 1–4 years (OR = 1.03 per 50 additional TKAs/year, 95% CI [0.97–1.09], 3 studies [4, 8, 74], *I*^2^ = 0%, very low certainty, Table 3). However, the sensitivity analysis comparing highest vs. lowest volume categories showed that higher hospital volume may be associated with a higher rate of deep infection (OR = 1.60; 95% CI [0.91–2.82], *I*^2^ = 54%, Supplementary Table 13).

#### Adverse events

Due to the heterogeneous clinical definitions of adverse events in the primary studies (Supplementary Table 3), this outcome was not pooled. The relationship between hospital volume and adverse event rates ≤ 3 months was inconsistent across studies in a narrative synthesis (Supplementary Tables 4, 9), and the certainty was very low based on 7 studies [52, 54, 60, 77, 94, 98, 118].

#### Readmission

A higher hospital volume was likely associated with a slightly lower readmission rate ≤ 3 months (OR = 0.98; 95% CI [0.97–0.99], 3 studies [7, 81, 122], *I*^*2*^ = 44%, moderate certainty, Table 3, Fig. 2b). The direction of this relationship was robust to sensitivity analyses (Supplementary Tables 13, 14), although the relationship was no longer statistically significant when only unadjusted effect estimates were included (Supplementary Table 16).

### Other secondary outcomes

Limited evidence (Supplementary Table 6) showed that higher hospital volume may be associated with lower rates of the following outcomes:

1. Composite adverse events including mortality ≤ 3 months [22, 40, 57, 98, 104],
2. Any infection ≤ 3 months [45, 98, 104, 118] and > 3 months [22, 54, 104]
3. Length of hospital stay [1, 32, 33, 45, 47, 51, 54, 60, 68, 76, 81, 83, 85, 110, 111, 118, 121],
4. Pneumonia ≤ 3 months [52],
5. Superficial infection ≤ 3 months [7, 49, 78] and > 3 months [3, 71, 101],
6. ‘Surgical complications’ as a composite outcome ≤ 3 months [18, 40, 47, 83, 94],
7. Thromboembolic events ≤ 3 months [45, 52, 98, 104] and > 3 months [104] and,
8. Thrombophlebitis ≤ 3 months [104] and > 3 months [104].

Hospital volume may be associated with function ≤ 3 months in a U-shaped relationship [42, 49]. Specifically, postoperative mobility at discharge appeared to be highest at hospital volumes of approximately 300–400 TKAs/year, and hospitals with lower or higher TKA volumes had worse outcomes [49].

There was no evidence for a relationship between hospital volume and the rates of the following outcomes:

1. Deep infection ≤ 3 months [52, 58],
2. Mortality > 3 months [22, 40, 57, 98, 104],
3. Myocardial infarction ≤ 3 months [17, 52, 98],
4. Quality of life > 3 months [115],
5. Readmission > 3 months [51] and
6. Wound haematoma or secondary haemorrhage ≤ 3 months [78].

Although patient satisfaction was reported in two studies [32, 92], we did not synthesise the results due to critical risk of bias.

### Certainty of evidence

Table 4 shows the GRADE assessment and summary of findings for the primary and main secondary outcomes. The individual GRADE domains and the certainty of evidence for the other secondary outcomes are shown in Supplementary Tables 5 and 6, respectively*.* The certainty of evidence was moderate for 4 outcomes, low for 7 outcomes, very low for 15 outcomes and not assessed for 1 outcome.

**Table 4:** Summary of findings and certainty of evidence (GRADE)

Number of studies	Study event rates	Effect		Certainty	Importance
	(*n/N*) [%]	Extreme comparison Relative [95% CI] Absolute [95% CI] Alternatively: SWiM	Dose–response *OR* per 50 TKAs/year increase [95% CI]	Certainty rating Reason for rating	
**Primary outcome: early revision (≤ 12 months)**					
7 studies in SWiM [5, 50, 54, 61, 65, 82, 83]	*N* = 301,378	In 5 studies accounting for 87% of patients, higher hospital volume was associated with lower rates of early revision		⊕⊕⚪ **Low** –2 for risk of bias	Critical
**Main secondary outcomes**					
Mortality (all cause, ≤ 3 months)					
9 studies in meta-analysis [45, 51, 52, 54, 60, 76, 83, 98, 104]	4769/2,638,996 (0.2%)	**OR 0.62** [0.48–0.79] **1 fewer per 1000** (from 1 to 0 fewer)	Linear dose–response gradient **OR 0.91 [0.87–0.95]**	⊕⊕⊕⚪ **Moderate** –1 for risk of bias –1 for inconsistency +1 for dose–response gradient	Critical
Infection (deep) (1–4 years)					
3 studies in meta-analysis [4, 8, 74]	797/97,019 (0.8%)	**OR 1.60** [0.91–2.82] **5 more per 1000** [from 1 fewer to 15 more]	No evidence for a dose–response association	⊕⚪⚪⚪ **Very low** –2 for risk of bias, –1 for imprecision	Critical
Revision (1–5 years)					
5 studies in meta-analysis [5, 50, 51, 54, 73]	5,498/163,520 (3.4%)	**OR 0.99** [0.65–1.50] **0 fewer per 1 000** [from 12 fewer to 16 more]	No evidence for a dose–response association	⊕⚪⚪⚪ **Very low** –2 for risk of bias, –1 for inconsistency, –1 for imprecision	Important
Adverse events (≤ 3 months)					
7 studies in SWiM [52, 54, 60, 77, 94, 98, 118]	*N* = 1,396,241	The effect of hospital volume on this composite outcome was inconsistent across studies		⊕⚪⚪⚪ **Very low** –2 for risk of bias, –1 for inconsistency	Important
Revision (6–10 years)					
5 studies in SWiM [5, 9, 30, 81, 97]	*N* = 684,733	Results were inconsistent across studies		⊕⚪⚪⚪ **Very low** –2 for risk of bias, –1 for inconsistency	Important
Readmission (≤ 3 months)					
3 studies in meta-analysis [7, 81, 122]	78,895/830,381 (9.5%)	**OR 0.85** [0.74–0.98] **13 fewer per 1000** [from 23 to 2 fewer]	Linear dose–response gradient, **OR 0.98** [0.97–0.99]	⊕⊕⊕⚪ **Moderate** –2 for risk of bias +1 for dose–response gradient	Important

*CI* confidence interval, *I*^*2*^ index for residual heterogeneity, *k* number of studies, *n* patients with event, *N* number of patients at risk, *OR* odds ratio, *ROBINS-I* risk of bias in non-randomised studies of interventions tool, *SWiM* synthesis without meta-analysis, *TKA* total knee arthroplasty

## Discussion

The current systematic review reports the results of a dose–response meta-analysis of 68 cohort studies that assessed the relationship between hospital TKA volume and patient-relevant outcomes. As hypothesised, higher hospital TKA volume may be associated with a lower rate of early revisions and is likely associated with small reductions in mortality and readmission ≤ 3 months after TKA. Earlier systematic reviews by Critchley [26] and Stengel [107] also found small reductions in mortality with increased hospital TKA volume, whereas Marlow [62] found no evidence for this association.

The certainty of evidence of the synthesised results was reduced by the relatively high risk of bias resulting from the observational design of the primary studies, which lies in the nature of the topic. Furthermore, the selection of endpoints for this systematic review was limited to morbidity and mortality, which are more widely recorded than outcomes related to function and quality of life. As a result, the association of hospital volume with improvements in function, quality of life, and pain reduction (the primary goals of TKA) could not be assessed. Mortality may not be the most relevant endpoint to study from a patient perspective, and overall event rates are very low. Nevertheless, the results may be may be clinically relevant at the population level.

Higher hospital volume does not directly result in improved patient outcomes but, rather, acts as a proxy measure for quality [66, 70]. Three general explanatory factors for the hospital volume–outcome relationship have been identified for various medical procedures: level of specialisation, hospital-level factors including nursing staff and facilities, and compliance with evidence-based processes [66]. In addition, there is a tendency for a surgeon volume–outcome relationship in TKA surgery [69]. Based on the results of this systematic review, surgeon volume could constitute one aspect of the hospital volume–outcome relationship, since the meta-analysis no longer showed a significant association with mortality when only data adjusted for surgeon volume were included (Supplementary Table 15). In several types of cancer surgeries and cardiovascular procedures, surgeon volume accounts for a large proportion of the effect of hospital volume [15]. Therefore, the authors interpret hospital volume as a proxy for quality, of which surgeon volume is one element. Additional confounders exist, e.g. patient characteristics [26] and changing suppliers of implant systems [105].

Understanding the volume–outcome relationship is important in light of discussions regarding the centralisation of surgical procedures to specialised hospitals [14, 62]. These results suggest that centralising TKA surgery may improve patient outcomes. A drawback of centralisation is that it may increase patients’ travel burden and reduce access for disadvantaged patients [14, 56, 66, 96].

Future studies should adhere to reporting guidelines [11, 117] so that their data can be used more effectively for further research. To evaluate whether the volume–outcome relationship for TKA is non-linear, a future primary study could use multinational registry data. Measurement of patient-reported outcomes in the context of the hospital volume–outcome relationship is desirable.

This systematic review has several limitations. First, the results are based on a relatively small number of studies for most outcomes, although a large number of studies were included in this systematic review. This was because primary studies did not report the same outcomes, and time points or data required for the dose–response meta-analysis were missing. Second, the small number of volume categories in the primary studies may have hidden non-linear relationships, which could therefore have gone undetected by a dose–response meta-analysis. Third, the applicability of the results to other healthcare systems is limited because a large proportion of data were collected in North America. Fourth, there was considerable between-study heterogeneity for most outcomes, probably due to inconsistent methodology in primary studies, variation among healthcare systems and regulatory approaches, and different periods of data collection. Sources of heterogeneity could not be explored by subgroup analysis because there were fewer than three studies per subgroup for each outcome. However, when the highest and lowest volume categories were compared, heterogeneity decreased, and pooled effect estimates showed strengthened associations between hospital volume and outcomes. Fifth, it was not possible to assess publication bias because fewer than ten studies per outcome were included in the dose–response meta-analyses [109]. Because of these limitations, conclusions should be drawn from the direction and dimensions of the hospital volume–outcome associations rather than the exact numerical values of the pooled effect sizes.

## Conclusion

Policy makers need solid evidence when regulating surgical procedures. The results for TKA show that there is moderate to low certainty evidence for an inverse hospital volume–outcome relationship for the outcomes of mortality, readmissions and early revisions. These small reductions in unfavourable outcomes may be clinically relevant at the population level. This finding supports the centralisation of TKA surgery to high-volume hospitals.

## Supplementary Information

Below is the link to the electronic supplementary material.

Supplementary file1 (DOCX 1461 KB)

Supplementary Table 4 (XLSX 524 KB)

Supplementary Table 9 (XLSX 486 KB)

## Data Availability

Additional details regarding methodology and data are available upon reasonable request from the corresponding author.

## Abbreviations

CI: Confidence interval
GRADE: Grading of recommendations, assessment, development, and evaluation
*k*: Number of studies
*n*: Patients with event (outcome)
*N*: Number of patients at risk
OR: Odds ratio
PRESS: Peer review of electronic search strategies
PRISMA: Preferred reporting items for systematic reviews and meta-analyses
ROBINS-I: Risk of bias in non-randomised studies of interventions
SWiM: Synthesis Without Meta-analysis
TKA: Total knee arthroplasty
