# Supplementary material for: Hospital volume–outcome relationship in total knee arthroplasty: a systematic review and dose–response meta-analysis
Source: Knee Surg Sports Traumatol Arthrosc. 2021 Sep 8;30(8):2862–77. doi: 10.1007/s00167-021-06692-8 (PMC9309153; doi:10.1007/s00167-021-06692-8)
Supplement: Supplementary file 1 — Supplementary file1 (DOCX 1461 KB) [file 167_2021_6692_MOESM1_ESM.docx]

Supplementary material

Hospital volume-outcome relationship in total knee arthroplasty: A systematic review and dose-response meta-analysis, Knee Surgery, Sports Traumatology, Arthroscopy

**C. M. Kugler, K. Goossen, T. Rombey, K. K. De Santis, T. Mathes, J. Breuing, S. Hess, R. Burchard, D. Pieper; DOI: 10.1007/s00167-021-06692-8**

Correspondence to Charlotte M. Kugler, Institute for Research in Operative Medicine (IFOM), Witten/Herdecke University, Ostmerheimer Str. 200, 51109 Cologne, Germany, [charlotte.kugler@uni-wh.de](mailto:charlotte.kugler@uni-wh.de)

**Contents**

[Supplementary Material 1. Electronic search terms and syntax 2](#_Toc79566285)

[Supplementary Material 2. Details of linear dose-response meta-analysis 5](#_Toc79566286)

[Supplementary Material 3. Sensitivity analyses and ‘best-available’ meta-analysis 6](#_Toc79566287)

[Supplementary Material 4. Synthesis Without Meta-Analysis Method 7](#_Toc79566288)

[Supplementary Table 1. Information sources 8](#_Toc79566289)

[Supplementary Table 2. Eligibility criteria 9](#_Toc79566290)

[Supplementary Table 3. Data items (outcome measures) extracted from the included studies 10](#_Toc79566291)

[Supplementary Table 5. GRADE assessment of primary and main secondary outcomes 12](#_Toc79566292)

[Supplementary Table 6. GRADE assessment other secondary outcomes 14](#_Toc79566293)

[Supplementary Table 7. Excluded studies based on full-text screening 19](#_Toc79566294)

[Supplementary Table 8. Study and patient characteristics, definition of volume categories, endpoints and conclusions for each included study 20](#_Toc79566295)

[Supplementary Table 10. Risk of bias assessment by outcome and data 25](#_Toc79566296)

[Supplementary Table 11. Results of linear dose-response meta-analysis of best-adjusted effect estimates (additional secondary outcomes) 34](#_Toc79566297)

[Supplementary Table 12. Results of linear dose-response meta-analysis of best-available effect estimates (adjusted and unadjusted) 35](#_Toc79566298)

[Supplementary Table 13. Sensitivity analysis 1: univariate meta-analysis of highest vs. lowest hospital volumes 36](#_Toc79566299)

[Supplementary Table 14. Sensitivity analysis 2: linear dose-response meta-analysis of effect estimates adjusted for age, gender and comorbidity 36](#_Toc79566300)

[Supplementary Table 15. Sensitivity analysis 3: linear dose-response meta-analysis of effect estimates adjusted for age, gender, comorbidity and surgeon volume 36](#_Toc79566301)

[Supplementary Table 16. Sensitivity analysis 4: linear dose-response meta-analysis of unadjusted effect estimates 37](#_Toc79566302)

[References 38](#_Toc79566303)

# Supplementary Material 1. Electronic search terms and syntax

| **Databases:** |
| --- |
| ***Medline via PubMed:*** |
| Search string: "Hospitals, High-Volume"[Mesh] OR "Hospitals, Low-Volume"[Mesh] OR *“high volume hospital”[tiab] OR “high volume hospitals”[tiab] OR “low volume hospital”[tiab] OR “low volume hospitals”[tiab]* OR “volume outcome”[tiab] OR “hospital volume”[tiab] OR “hospital volumes”[tiab] OR “hospital size”[tiab] OR “clinic size”[tiab] OR “center volume”[tiab] OR “center volumes”[tiab] OR “centre volume”[tiab] OR “centre volumes”[tiab] OR “center size”[tiab] OR “centre size”[tiab] OR “patient volume”[tiab] OR “patient volumes”[tiab] OR “provider volume”[tiab] OR “provider volumes”[tiab] OR “surgical volume”[tiab] OR “surgical volumes”[tiab] OR “procedure volume”[tiab] OR “procedure volumes”[tiab] OR “procedural volume”[tiab] OR “procedural volumes”[tiab] OR “facility volume”[tiab] OR “facility volumes”[tiab] OR “treatment volume”[tiab] OR “treatment volumes”[tiab] OR regionali*[tiab] OR centrali*[tiab] OR decentrali*[tiab] OR caseload*[tiab] OR workload*[tiab] OR experience[tiab] OR performance[tiab]  AND ("Knee"[Mesh] OR "Arthroplasty, Replacement, Knee"[Mesh] OR "Osteoarthritis, Knee"[Mesh] OR *“knee replacement”[tiab] OR “knee replacements”[tiab]* OR arthroplasty[tiab] OR arthroplasties[tiab] OR TKA[tiab] OR osteoarthritis[tiab])  NOT ("Comment" [Publication Type] OR "Letter" [Publication Type] OR "Editorial" [Publication Type])  **Date of search and number of hits: 12.02.2020, 9,848** |
| ***Embase via Elsevier:*** |
| Search string: 'high volume hospital'/exp OR 'low volume hospital'/exp OR *'hospital volume'/exp* OR (“high volume hospital” OR “high volume hospitals” OR “low volume hospital” OR “low volume hospitals” OR “volume outcome” OR “hospital volume” OR “hospital volumes” OR “hospital size” OR “clinic size” OR “center volume” OR “center volumes” OR “centre volume” OR “centre volumes” OR “center size” OR “centre size” OR “patient volume” OR “patient volumes” OR “provider volume” OR “provider volumes” OR “surgical volume” OR “surgical volumes” OR “procedure volume” OR “procedure volumes” OR “procedural volume” OR “procedural volumes” OR “facility volume” OR “facility volumes” OR “treatment volume” OR “treatment volumes” OR regionali* OR centrali* OR decentrali* OR caseload* OR workload* OR experience OR performance):ti,ab,kw  AND ('knee'/exp OR 'knee replacement'/exp OR 'knee osteoarthritis'/exp OR (“knee replacement” OR “knee replacements” OR arthroplasty OR arthroplasties OR TKA OR osteoarthritis):ti,ab,kw)  AND ([embase]/lim)  NOT (('comment' OR 'letter' OR 'editorial'):it)  AND (embase NOT (embase AND medline)) |
| **Date of search and number of hits: 12.02.2020, 1,910** |
| ***CENTRAL via Cochrane Library:*** |
| Search string:  #1 MeSH descriptor: [Hospitals, High-Volume] explode all trees  #2 MeSH descriptor: [Hospitals, Low-Volume] explode all trees  #3 (“high volume hospital” OR “high volume hospitals” OR “low volume hospital” OR “low volume hospitals” OR “volume outcome” OR “hospital volume” OR “hospital volumes” OR “hospital size” OR “clinic size” OR “center volume” OR “center volumes” OR “centre volume” OR “centre volumes” OR “center size” OR “centre size” OR “patient volume” OR “patient volumes” OR “provider volume” OR “provider volumes” OR “surgical volume” OR “surgical volumes” OR “procedure volume” OR “procedure volumes” OR “procedural volume” OR “procedural volumes” OR “facility volume” OR “facility volumes” OR “treatment volume” OR “treatment volumes” OR regionali* OR centrali* OR decentrali* OR caseload* OR workload* OR experience OR performance):ti,ab,kw  #4 #1 OR #2 OR #3  #5 MeSH descriptor: [Knee] explode all trees  #6 MeSH descriptor: [Arthroplasty, Replacement, Knee] explode all trees  #7 MeSH descriptor: [Osteoarthritis, Knee] explode all trees  #8 (“knee replacement” OR “knee replacements” OR arthroplasty OR arthroplasties OR "TKA" OR osteoarthritis):ti,ab,kw  #9 #5 OR #6 OR #7 OR #8  #10 #4 AND #9  #11 (clinicaltrials.gov):so  #12 #10 NOT #11  **Date of search and number of hits: 12.02.2020, 1,654** |
| ***CINAHL via EBSCO:*** |
| Search string: ( ( TI (“high volume hospital” OR “high volume hospitals” OR “low volume hospital” OR “low volume hospitals” OR “volume outcome” OR “hospital volume” OR “hospital volumes” OR “hospital size” OR “clinic size” OR “center volume” OR “center volumes” OR “centre volume” OR “centre volumes” OR “center size” OR “centre size” OR “patient volume” OR “patient volumes” OR “provider volume” OR “provider volumes” OR “surgical volume” OR “surgical volumes” OR “procedure volume” OR “procedure volumes” OR “procedural volume” OR “procedural volumes” OR “facility volume” OR “facility volumes” OR “treatment volume” OR “treatment volumes” OR regionali* OR centrali* OR decentrali* OR caseload* OR workload* OR experience OR performance) OR AB (“high volume hospital” OR “high volume hospitals” OR “low volume hospital” OR “low volume hospitals” OR “volume outcome” OR “hospital volume” OR “hospital volumes” OR “hospital size” OR “clinic size” OR “center volume” OR “center volumes” OR “centre volume” OR “centre volumes” OR “center size” OR “centre size” OR “patient volume” OR “patient volumes” OR “provider volume” OR “provider volumes” OR “surgical volume” OR “surgical volumes” OR “procedure volume” OR “procedure volumes” OR “procedural volume” OR “procedural volumes” OR “facility volume” OR “facility volumes” OR “treatment volume” OR “treatment volumes” OR regionali* OR centrali* OR decentrali* OR caseload* OR workload* OR experience OR performance) ) AND ( MH "Knee" OR MH "Arthroplasty, Replacement, Knee" OR MH "Osteoarthritis, Knee" OR TI (“knee replacement” OR “knee replacements” OR arthroplasty OR arthroplasties OR TKA OR osteoarthritis) OR AB (“knee replacement” OR “knee replacements” OR arthroplasty OR arthroplasties OR TKA OR osteoarthritis) ) ) NOT ( PT ("Comment" OR "Letter" OR "Editorial") |
| **Date of search and number of hits: 12.02.2020, 3,825** |

# Supplementary Material 2. Details of linear dose-response meta-analysis

Studies with the following data were included in the meta-analysis:

1. Hospital volumes (midpoint of the standardised category)
2. Number of patients per hospital volume category
3. Number of patients with event per hospital volume category
4. Effect measure for the outcome (*OR*) at each hospital volume relative to the lowest hospital volume category expressed as a natural logarithm
5. 95% *CI* corresponding to the effect measure for the outcome (*OR*) at each hospital volume relative to the lowest hospital volume category.

For the dose-response meta-analysis, we estimated a curve for each study across hospital volume values and pooled these curves into an overall hospital volume-outcome curve [167]. Based on pooled curves, we calculated outcome-specific pooled *ORs* per 50 additional TKA per year and 95% *CIs*. In the case non-linearity would had been detected, we planned a non-linear dose-response meta-analysis [206]*.* Volume categories with no patients with event were excluded from the analysis. This was done because *ORs* could not be computed for null categories [224]. In addition, there were some studies reporting data for individual hospitals. If all hospitals were classified into the same volume category according to our classification (1-24, 25-49, 50-74, 75-99, 100-124, 125-150, 150+; e.g. [191]), we excluded these studies from meta-analysis. This was done because such studies used a single volume category according to our classification.

# Supplementary Material 3. Sensitivity analyses and ‘best-available’ meta-analysis

We conducted three sensitivity analyses planned *a priori* and one *post hoc*. The first was a univariate inverse-variance random-effects meta-analysis comparing highest vs. lowest volume categories. We used the Paule and Mandel heterogeneity variance estimator and modified Hartung-Knapp *CIs* for the pooled estimates [113, 245]. Beta-binomial models (random-effects model) were computed for rare events, such as mortality [159] (results reported in Supplementary Table 13). The second sensitivity analysis included only studies adjusted at least for age, gender and comorbidity (results reported in Supplementary Table 14), the third included only studies adjusted additionally for surgeon volume (results reported in Supplementary Table 15), and the forth (*post-hoc*) included only unadjusted *OR*s (results reported in Supplementary Table 16).

We conducted one additional dose-response meta-analysis (*post-hoc*) using ‘best available’ effect estimates. These included any available data per outcome, ideally the ‘best adjusted’ effect estimates used in the main meta-analysis or, if adjusted data were unavailable, unadjusted effect estimates (results reported in Supplementary Table 12).

Included studies: included studies[4, 9, 10, 12-15, 17, 18, 32, 34, 35, 37, 46, 49, 55, 56, 60, 62, 64, 71, 79, 80, 86, 88, 93, 94, 98, 100-102, 110, 112, 121, 124, 126, 127, 136, 146, 147, 161, 163, 168, 170, 171, 176, 178, 179, 183-185, 187, 188, 190, 191, 193, 198, 200, 211, 214, 217, 221, 224, 228, 229, 231, 233, 234, 237, 238, 240, 243, 244, 251, 252, 254, 256, 257]

# Supplementary Material 4. Synthesis Without Meta-Analysis Method

We followed the guideline by Campbell et al. [43].

For narrative synthesis, studies reporting both adjusted and unadjusted effect measures were combined.

When it was not possible to undertake a meta-analysis of effect estimates due to missing data or a low number of contributing studies, we synthesised the results without meta-analysis. All available, non-overlapping studies for such outcomes were used. We based our conclusions on patient numbers contributing to the outcome.

We assessed consistency by comparing the direction of effect between studies. Results were considered inconsistent if the direction of effect varied across studies, or when the direction of effect varied within study subgroups. Results were considered consistent if the same trend was observed for studies covering a large proportion (80% or more) of patients.

We assessed indirectness when the population was substantially different to our research question, e.g. if only patients with diabetes having TKA were included.

When it was not possible to undertake a meta-analysis of effect estimates, we assessed imprecision by considering confidence intervals for the largest studies, covering more than half the included patients.

The certainty of the narrative synthesis findings was assessed using GRADE, specifically using guidelines on applying the GRADE approach when evidence for an effect is summarised narratively [177].

# Supplementary Table 1. Information sources

| **Type** | **Source** | **Timeframe** |
| --- | --- | --- |
| Electronic databases | MEDLINE via PubMed  EMBASE via EMBASE  Cochrane Central Register of Controlled Trials (CENTRAL) via Cochrane Library  CINAHL via EBSCO | Inception to 12.02.2020 |
| Trial registers | ClinicalTrials.gov  German Clinical Study Register (DRKS)  International Clinical Trials Registry Platform (ICTRP) | Inception to 12.02.2020 |
| Conference proceedings online | International Society of Arthroscopy, Knee Surgery and Orthopaedic Sports Medicine (ISAKOS)  American Academy of Orthopaedic Surgeons (AAOS)  European Knee Society (EKS)  Pan Pacific Orthopeadic Congress  Société Internationale de Chirurgie Orthopédique et de Traumatologie (SICOT)  American Orthopaedic Society for Sports Medicine (AOSSM) | 2003-2017  2014-2019  No complete summary of conference proceedings was publicly available. The EKS published selected proceedings from 2017-2018 (n=5) in 09/2019 in *Clinical Orthopaedics and Related Research* which we would have found by our update search if they were relevant.  2014-2016  No conference proceedings publicly available  2013-2019 |
| Other sources | Manual search of reference lists of all included primary studies in our and other systematic reviews.  Forward-citation search (Web of Science)  Expert contact for additional studies (six corresponding authors of other systematic reviews). | Inception to 26.03.2020 |

# Supplementary Table 2. Eligibility criteria

| **Inclusion criteria** | **Exclusion criteria** |
| --- | --- |
| Primary studies   1. with the following PICO criteria: 2. **Population**: Patients with primary and/or revision TKA 3. **Intervention/Control**: TKA conducted at hospitals with at least two different hospital volumes (reported as volume categories or continuous values) 4. **Outcome**: At least one patient-relevant outcome 5. that report results for TKA patients separately from other surgical procedures 6. published in peer-reviewed journals or conference abstracts or unpublished (registered trials) 7. with the following designs: (cluster-) RCTs and prospective or retrospective cohort studies 8. available as full-text | 1. Studies analysing data from one hospital only 2. Studies analysing only the influence of surgeon volume but not hospital volume 3. Modelling studies 4. Additions to the protocol: 5. Studies using volume categories from revision TKA instead of total TKA. This was done because the revision volumes were not comparable to overall hospital TKA volumes. 6. Studies comparing less than two hospitals in the same country / healthcare system. This was done because differences in the healthcare systems may have a higher impact on outcomes than hospital volumes. 7. Studies comparing hospital volume-outcome relationships within different time periods. This was done to avoid potential time-varying confounding. |

Note. Studies excluded for the reasons a)-c) are listed in *Table vii*. Abbreviation: PICO, Patient, Intervention, Control, Outcome; RCT, randomised controlled trial; TKA, total knee arthroplasty.

# Supplementary Table 3. Data items (outcome measures) extracted from the included studies

| **Outcome** | **Definition** | **Time of measurement** |
| --- | --- | --- |
| **Primary Outcome** | | |
| Early Revision | any procedure involving removal, exchange or addition of any implant part caused for example by infection or aseptic mechanical failure | ≤12 months |
| **Secondary Outcomes** | | |
| Revision | any procedure involving removal, exchange or addition of any implant part caused for example by infection or aseptic mechanical failure | 1-5 years, 6-10 years |
| Mortality | any-cause death within or after the patient’s index hospital stay | ≤3 months;  >3 months |
| Infection (deep) | periprosthetic joint infection requiring any appropriate surgical procedure such as total joint revision surgery, debridement, tibial liner removal, arthrotomy, debridement, synovectomy, or above-the-knee amputation after the index operation.  also includes revision due to infection[10] | ≤3 months;  >3 months |
| Adverse events | various definitions across studies including accidental surgical mishaps, cardiovascular events (myocardial infarction, cardiac dysrhythmias, stroke, pulmonary embolism, thrombosis, pulmonary embolism), pneumonia, upper gastrointestinal bleeding, disorders of fluid, electrolyte, and acid-base balance, complications affecting specified body systems, rupture of tendon, urinary tract infection **but no deaths** | ≤3 months |
| Readmission | all-cause hospital readmission after discharge following the hospital stay associated with the patient's index TKA | ≤3 months;  >3 months |
| Infection (any) | postoperative infections that were not further specified into superficial or deep infections | ≤3 months;  >3 months |
| Infection (superficial) | postoperative skin, superficial or subcutaneous wound infections | ≤3 months;  >3 months |
| Wound complications | wound hematoma or secondary haemorrhage | ≤3 months |
| Surgical complications composite | any postoperative complication related to the TKA within or after index hospital stay, for example wound infection, abscess, haematoma, postoperative bleeding, mechanical complication of internal joint prosthesis, disruption of operation wound | ≤3 months |
| Thromboembolic event | included deep vein thrombosis, pulmonary embolism, venous thromboembolism | ≤3 months;  >3 months |
| Thrombophlebitis | any-cause thrombophlebitis (an infection of the veins causing thrombosis) | ≤3 months;  >3 months |
| Pneumonia | pneumonia requiring hospitalisation | ≤3 months |
| Myocardial infection | acute myocardial infection or other cardiac events (acute cardiac ischemia, arrhythmia) | ≤3 months |
| ‘Composite adverse events including mortality’ | various definitions across studies including all-cause postoperative complications, wound infection, deep infection, iatrogenic complications, ulceration, pulmonary compromise, acute myocardial infarction, venous thrombosis, pulmonary embolism, pneumonia, stroke, sepsis, septicemia, major bleeding, intracranial injury, burns, retained foreign object, air embolism, blood incompatibility, vascular catheter-associated infection, other complications (nervous system, peripheral vascular, respiratory, digestive system), gastrointestinal hemorrhage, prosthetic device malfunction  prolonged hospital stay, all-cause readmissions, urinary tract infections and complications, ileus **and death** | ≤3 months |
| Length of stay (LOS) | Mean or median length of stay in the hospital in days (from the date of admission to the date of discharge) for TKA | (duration) |
| Health-related quality of life | measured by EQ-5D-3L (EuroQol five-dimensional each with three levels of problems [244]) aggregate index scores, where 1 is defined as perfect health and 0 is defined as equivalent to being dead | >3 months |
| Function | measured by insufficient mobility defined as not reaching at least 90% knee bending and full extension of the knee at the time of hospital discharge; SF-36 (improvement in the physical composite score after knee replacement), Oxford Knee Score (overall score from 12 questions that reflect limitations in health-related quality of life brought about by the knee joint) | ≤3 months |
| Satisfaction | measured with satisfaction with the hospital (rated on a six-point scale) or Hospital Consumer Assessment of Healthcare Providers and Systems (HCAHPS) scoring (overall rating) | 1-25 months |

# Supplementary Table 5. GRADE assessment of primary and main secondary outcomes

| *Certainty assessment* | | | | | | | *Study event rates* | *Effect* |  | *Certainty* | *Importance* |
| --- | --- | --- | --- | --- | --- | --- | --- | --- | --- | --- | --- |
| Number of studies | Study design | Risk of bias | Inconsistency | Indirectness | Imprecision | Other considerations | (*n/N*) [%] | Extreme comparison  Relative [95% *CI*]  Absolute [95% *CI*]  alternatively: SwiM | Dose-response *OR* per 50 TKAs/year increase [95% *CI*] | Certainty Rating  Reason for rating |  |
| *Primary Outcome:* Early revision (≤12 months) | | | | | | | | | | | |
| 7 studies in SWiM [13, 121, 136, 163, 168, 191, 193] | observational studies | very serious^a^ | not serious^b^ | not serious | not serious^c^ | none | *N* = 301,378 | In 5 studies accounting for 87% of patients, higher hospital volume was associated with lower rates of early revision. | | ⊕⊕◯◯ LOW | CRITICAL |
| *Main Secondary Outcomes* | | | | | | | | | | | |
| Mortality (all cause, ≤3 months) | | | | | | | | | | | |
| 9 studies in meta-analysis [102, 124, 126, 136, 161, 184, 193, 224, 234] | observational studies | serious^d^ | serious^e^ | not serious | not serious ^f^ | dose response gradient | 4,769 / 2,638,996 (0.2 %) | ***OR* 0.62** [0.48 to 0.79]  **1 fewer per 1.000** [from 1 fewer to 0 fewer] | linear dose-response gradient,  ***OR* 0.91**  [0.87 to 0.95] | ⊕⊕⊕◯  MODERATE | CRITICAL |
| Infection (deep) (1 to 4 years; deep infection / revision due to deep infection) | | | | | | | | | | | |
| 3 studies in meta-analysis [12, 17, 179] | observational studies | very serious^g^ | not serious | not serious | serious^h^ | none | 797 / 97,019 (0.8 %) | ***OR* 1.60** [0.91 to 2.82]  **5 more per 1.000** [from 1 fewer to 15 more] | No evidence for a dose-response association | ⊕◯◯◯ VERY LOW | CRITICAL |
| Revision (1 to 5 years) | | | | | | | | | | | |
| 5 studies in meta-analysis [13, 121, 124, 136, 178] | observational studies | very serious^a^ | serious^i^ | not serious | serious^h^ | none | 5,498 / 163,520 (3.4 %) | ***OR* 0.99** [0.65 to 1.50]  **0 fewer per 1.000** [from 12 fewer to 16 more] | No evidence for a dose-response association. | ⊕◯◯◯ VERY LOW | IMPORTANT |
| Adverse events (≤3 months) | | | | | | | | | | | |
| 7 studies in SWiM [126, 136, 161, 185, 217, 224, 251] | observational studies | very serious^a^ | serious ^k, l^ | not serious | not serious^m^ | none | *N* = 1,396,241 | The effect of hospital volume on this composite outcome was inconsistent across studies. | | ⊕◯◯◯ VERY LOW | IMPORTANT |
| Revision (6 to 10 years) [163] out for overlap with [71] | | | | | | | | | | | |
| 5 studies in SWiM [13, 18, 71, 190, 221] | observational studies | very serious^g^ | serious^k^ | not serious | not serious^n^ | none | *N* = 684,733 | Results were inconsistent across studies. | | ⊕◯◯◯ VERY LOW | IMPORTANT |
| Readmission (all cause, ≤3 months) | | | | | | | | | | | |
| 3 studies in meta-analysis [15, 190, 257] | observational studies | very serious^a^ | not serious | not serious | not serious^f^ | dose response gradient | 78,895 / 830,381 (9.5 %) | ***OR* 0.85** [0.74 to 0.98]  **13 fewer per 1.000** [from 23 fewer to 2 fewer] | linear dose-response gradient,  ***OR* 0.98**  [0.97 to 0.99] | ⊕⊕⊕◯ MODERATE | IMPORTANT |

Note. *CI* = confidence interval; *I^2^* = index for residual heterogeneity; MA = meta-analysis; n = patients with event; *N =* number of patients at risk; *OR =* odds ratio; ROBINS-I = Risk Of Bias In Non-randomized Studies of Interventions tool; SwiM = Synthesis without meta-analysis TKA = total knee arthroplasty.

a. Overall risk of bias was serious in all but one study, and moderate in one study.

b. Same trend for 5 studies accounting for >80% of patients, one study with opposite effect, and one study included too few hospitals. In 4 individual studies there was a consistent decrease with increasing hospital volumes.

c. Some studies, including the study with the largest number of patients, reported small reductions, and other studies reported ‘non-significant results’. Borderline decision not to downgrade.

d. Overall risk of bias was serious in 5 studies and moderate in 4 studies. Since studies with moderate risk of bias accounted for >80% of patients and events, and dominated the results we assume that the overall result is not seriously biased.

e. Significant residual heterogeneity

f. Narrow confidence interval, excluded no effect. Result robust across sensitivity analyses.

g. Overall risk of bias was serious in all studies.

h. Confidence interval includes both a positive and a negative effect of increasing hospital volume on outcome and fails to exclude important benefits or harms.

i. Substantial unexplained heterogeneity beyond that expected by clinical differences in different health-care systems (*I*^2^ was >75% in main meta-analysis and all sensitivity analyses).

k. The direction of effect varied across studies.

l. Substantial differences in outcomes measures, resulting from the diversity of definitions for this composite outcome. Studies not pooled.

m. Confidence interval of the largest study excluded no effect.

n. Most studies found an effect for some but not all volume categories.

# Supplementary Table 6. GRADE assessment other secondary outcomes

| *Certainty assessment* | | | | | | | | *Study event rates* | | *Effect* | | |  | | *Certainty* | | *Importance* | |  |
| --- | --- | --- | --- | --- | --- | --- | --- | --- | --- | --- | --- | --- | --- | --- | --- | --- | --- | --- | --- |
| Number of studies | Study design | Risk of bias | Inconsistency | Indirectness | Imprecision | Other considerations | (*n/N*) [%] | | Extreme comparison  Relative [95% *CI*]  Absolute [95% *CI*]  alternatively: SWiM | | | Dose-response *OR* per 50 TKAs/year increase [95% *CI*] | | Certainty Rating | |  | |  |  |
| ‘Composite adverse events including mortality’ ≤3 months | | | | | | | | | | | | | | | | | | | |
| 6 studies in SWiM [79, 80, 86, 168, 228, 256]  (*[254] out for overlap with [86])* | observational studies | very serious^[[1]](#endnote-2)^ | not serious^[[2]](#endnote-3)^ | not serious | not serious^[[3]](#endnote-4)^ | none | *N* = 847,994 | | In 4 studies accounting for 95% of patients, higher hospital volume was associated with lower rates of adverse events (including mortality). | | | | | ⊕⊕◯◯ LOW | | CRITICAL | |  |  |
| Infection (any) ≤3 months | | | | | | | | | | | | | | | | | | | |
| 4 studies in MA [102, 224, 234, 251] | observational studies | very seriousa | serious^[[4]](#endnote-5)^ | not serious | not serious | none | 1,976 / 329,280 (0.6 %) | | ***OR* 0.57** [0.44 to 0.74]  **3 fewer per 1.000** [from 3 fewer to 2 fewer] | | | No evidence for a dose-response association. | | ⊕◯◯◯ VERY LOW | | IMPORTANT | |  |  |
| Infection (any) >3 months | | | | | | | | | | | | | | | | | | | |
| 3 studies in SWiM [49, 136, 234] | observational studies | serious^[[5]](#endnote-6)^ | not seriousb | not serious | not serious (borderline)^[[6]](#endnote-7)^ | none | *N* = 269,236 | | In all studies higher hospital volume was associated with lower rates of infection. | | | | | ⊕⊕⊕◯ MODERATE | | IMPORTANT | |  |  |
| Infection (deep) ≤3 months | | | | | | | | | | | | | | | | | | | |
| 2 studies in SWiM [126, 147] | observational studies | serious^[[7]](#endnote-8)^ | very serious^[[8]](#endnote-9)^ | not serious | not serious (borderline)f | none | *N* = 651,139 | | Results were inconsistent across studies; higher hospital volume was associated with higher rates of infection in the larger study with lower risk of bias. | | | | | ⊕◯◯◯ VERY LOW | | CRITICAL | |  |  |
| Function ≤3 months | | | | | | | | | | | | | | | | | | | |
| 2 studies in SWiM [98, 112] | observational studies | seriousg | not serious^[[9]](#endnote-10)^ | not serious | not serious^[[10]](#endnote-11)^ | none | *N* = 110,640 | | In one study accounting for >99% of patients, hospital volume was associated with insufficient postoperative mobility in a U-shaped curve with a minimum around 300-400 TKA/y. | | | | | ⊕⊕⊕◯ MODERATE | | NOT IMPORTANT^[[11]](#endnote-12)^ | |  |  |
| Length of stay *([191] out for overlap with [190])* | | | | | | | | | | | | | | | | | | | |
| 17 studies in SWiM [4, 79, 80, 102, 110, 124, 136, 161, 171, 184, 190, 193, 198, 237, 238, 251, 256] | observational studies | very seriousa | serious^[[12]](#endnote-13)^ | not serious | not seriousj | none | *N* = 2,621,473 | | Length of stay tended to be longer for hospital volume below about 50-150 TKA/year, in studies accounting for 94% of patients. The direction of effect was opposite in Japan. | | | | | ⊕◯◯◯ VERY LOW | | NOT IMPORTANT | |  |  |
|  |  |  |  |  |  |  |  | |  | | | | |  | |  | |  |  |
| Mortality >3 months | | | | | | | | | | | | | | | | | | | |
| 5 studies in SWiM [49, 94, 146, 224, 234] | observational studies | very seriousa | seriousl | not serious | not seriousj | none | *N* = 347,931 | | Higher hospital volume was associated with either lower rates of mortality or no difference in outcome. | | | | | ⊕◯◯◯ VERY LOW | | CRITICAL | |  |  |
| Myocardial infarction ≤3 months | | | | | | | | | | | | | | | | | | | |
| 3 studies in SWiM [35, 126, 224] | observational studies | very seriousa | not serious | not serious | not serious^[[13]](#endnote-14)^ | none | *N* = 338,695 | | No association between hospital volume and myocardial infarction rates was observed. | | | | | ⊕⊕◯◯ LOW | | IMPORTANT | |  |  |
| Pneumonia ≤3 months | | | | | | | | | | | | | | | | | | | |
| 1 study in SWiM [126] | observational study | very serious^[[14]](#endnote-15)^ | not serious | not serious | seriousv | none | *N* = 78,824 | | Higher hospital volume was associated with lower rates of pneumonia in a single study. | | | | | ⊕◯◯◯ VERY LOW | | IMPORTANT | |  |  |
| Quality of Life >3 months | | | | | | | | | | | | | | | | | | | |
| 1 study in SWiM [244] | observational study | very serious | not serious | not serious | seriousv | none | *N* = 83,648 | | Hospital volume was not associated with patients’ reports of changes to their disease-specific or generic HRQL in a single study. | | | | | ⊕◯◯◯ VERY LOW | | IMPORTANT | |  |  |
| Readmission >3 months | | | | | | | | | | | | | | | | | | | |
| 1 study in SWiM [124] | observational study | very serious | not serious | not serious | serious^[[15]](#endnote-16)^ | none | *N* = 34,567 | | No clear trend was observed. | | | | | ⊕◯◯◯ VERY LOW | | NOT IMPORTANT | |  |  |
| Satisfaction between discharge and 2 years after surgery | | | | | | | | | | | | | | | | | | | |
| Patient satisfaction was not synthesised because this outcome was at critical risk of bias for all studies [79, 211]. | | | | | | | | | | | | | | | | | IMPORTANT | |  |
| Infection (superficial) ≤3 months | | | | | | | | | | | | | | | | | | | |
| 3 studies in SWiM [15, 112, 187] | observational studies | very serious^[[16]](#endnote-17)^ | not serious | not serious | not serious | none | *N* = 1,184,454 | | In all studies higher hospital volume was associated with lower rates of superficial infection. | | | | | ⊕⊕◯◯ LOW | | IMPORTANT | |  |  |
| Infection (superficial) >3 months | | | | | | | | | | | | | | | | | | | |
| 3 studies in MA [10, 176, 229] | observational studies | very serious^[[17]](#endnote-18)^ | seriousd | not serious | serious^[[18]](#endnote-19)^ | none | 558 / 22,324 (2.5 %) | | ***OR* 0.67** [0.24 to 1.89]  **8 fewer per 1.000** [from 19 fewer to 21 more] | | No evidence for a dose-response association. | | | ⊕◯◯◯ VERY LOW | | IMPORTANT | |  |  |
| Surgical complications composite ≤3 months | | | | | | | | | | | | | | | | | | | |
| 5 studies in SWiM [37, 94, 110, 193, 217] | observational studies | very seriousq | serious^[[19]](#endnote-20)^ | not serious | serious^[[20]](#endnote-21)^ | none | *N* = 441,098 | | In three studies accounting for 93% of patients, higher hospital volume was associated with lower rates of surgical complications. | | | | | ⊕◯◯◯ VERY LOW | | CRITICAL | |  |  |
| Thromboembolic events ≤3 months | | | | | | | | | | | | | | | | | | | |
| 4 studies in MA [102, 126, 224, 234] | observational studies | very seriousa | not serious | not serious | seriousr | none | 1,974 / 376,486 (0.5 %) | | ***OR* 0.82** [0.50 to 1.34]  **1 fewer per 1.000** [from 3 fewer to 2 more] | | No evidence for a dose-response association. | | | ⊕◯◯◯ VERY LOW | | CRITICAL | |  |  |
| Thromboembolic events >3 months | | | | | | | | | | | | | | | | | | | |
| 1 study in SWiM [234] | observational study | serious^[[21]](#endnote-22)^ | not serious | not serious | serious^[[22]](#endnote-23)^ | none | *N* = 222,684 | | Higher hospital volume was associated with lower rates of pulmonary embolism in a single study. | | | | | ⊕⊕◯◯ LOW | | IMPORTANT | |  |  |
| Thrombophlebitis ≤3 months | | | | | | | | | | | | | | | | | | | |
| 1 study in SWiM [234] | observational study | seriousu | not serious | not serious | seriousv | none | *N* = 222,684 | | Higher hospital volume was associated with lower rates of thrombophlebitis in a single study. | | | | | ⊕⊕◯◯ LOW | | IMPORTANT | |  |  |
| Thrombophlebitis >3 months | | | | | | | | | | | | | | | | | | | |
| 1 study in SWiM [234] | observational study | seriousu | not serious | not serious | seriousv | none | *N* = 222,684 | | Higher hospital volume was associated with lower rates of thrombophlebitis in a single study. | | | | | ⊕⊕◯◯ LOW | | NOT IMPORTANT | |  |  |
| Wound hematoma or secondary haemorrhage ≤3 months | | | | | | | | | | | | | | | | | | | |
| 1 study in SWiM [187] | observational study | seriousu | serious^[[23]](#endnote-24)^ | not serious | seriousv | none | *N* = 354,593 | | Higher hospital volume was associated with less wound complications in one year but not in two other years studied. | | | | | ⊕◯◯◯ VERY LOW | | IMPORTANT | |  |  |

Note. *CI* = confidence interval; *I^2^* = index for residual heterogeneity; MA = meta-analysis; n = patients with event; *N =* number of patients at risk; *OR =* odds ratio; ROBINS-I = Risk Of Bias In Non-randomized Studies of Interventions tool; SwiM = Synthesis without meta-analysis TKA = total knee arthroplasty.

# Supplementary Table 7. Excluded studies based on full-text screening

| **Reason for exclusion** | **References** | **Number of studies** |
| --- | --- | --- |
| no TKA patients | [1, 16, 19, 20, 22, 26, 28, 31, 36, 38, 48, 52, 53, 59, 70, 72, 82, 83, 87, 91, 96, 103-106, 120, 122, 123, 133, 143-145, 148, 162, 169, 173, 175, 181, 189, 192, 197, 199, 201, 204, 219, 223, 230, 235, 253] | 49 |
| no hospital volumes as exposure variable | [2, 3, 7, 8, 11, 21, 23, 29, 30, 39-42, 44, 45, 47, 50, 51, 54, 57, 61, 67, 73-78, 84, 89, 90, 92, 97, 99, 107, 109, 111, 114-117, 128-132, 134, 135, 137-141, 149-151, 153, 158, 160, 166, 172, 174, 186, 194, 196, 202, 203, 205, 207-209, 216, 220, 222, 225, 227, 241, 242, 246-249, 255, 258-260] | 86 |
| no patient-relevant outcomes | [5, 25, 27, 33, 65, 66, 85, 118, 154, 155, 157, 182, 232, 239, 250] | 15 |
| methodology study or review | [6, 58, 63, 69, 108, 119, 125, 132, 142, 156, 164, 165, 180, 195, 210, 212, 213, 215, 218, 226, 236] | 21 |
| Forward citation search (WoS): FT excluded |  | 95 |
| Near misses |  | 5 |
| 1. Studies using revision TKA volume categories. These were excluded because the revision volumes were not comparable to overall hospital TKA volumes. | [81, 95, 152] |  |
| 1. Studies comparing less than two hospitals in the same country / healthcare system. These were excluded because differences in the healthcare systems may have a higher impact on outcomes than hospital volumes. | [68] |  |
| 1. Studies comparing hospital volume-outcome relationships within different time periods. These were excluded to avoid potential time-varying confounding. | [24] |  |

# Supplementary Table 8. Study and patient characteristics, definition of volume categories, endpoints and conclusions for each included study

|  | ***Study characteristics*** | |  |  |  | ***Patients’ characteristics*** | | | ***Volume categories (per year)*** | | ***Results*** |  |
| --- | --- | --- | --- | --- | --- | --- | --- | --- | --- | --- | --- | --- |
| **Study (ref.)** | **Type of funding** | **Country (Region)** | **Primary data source** | **Data coll. (years)** | **No. of hospitals** | **No. of patients** | **% Female** | **Age (years)** | **Type** | **Upper limits; lower limit of highest category** | **Patient-relevant study outcomes** | **Authors’ conclusions favour** |
| Adhia 2019 [4] | none | USA (IL) | admin. | 2016–2018 | 151 | 72,359 | 63% | ≥65: 59% | thresholds | 200; 400; 600; ≥601 | LOS | higher volume |
| Amato 2017 [9] | non-profit | Italy | admin. | 2015–2015 | 690 | 71,838 | n.r. | n.r. | – (curve) | – | readmission | higher volume |
| Anis 2019 [10, 12] | n.r. | USA (OH, FL) | clinical | 2014–2017 | 16 | 12,541 | 62% | mean ± SD: 69 ± 10 | thresholds | 249; 500; ≥501 | infection | no evidence for a difference |
| Arias-de la Torre 2019 [13, 14] | non-profit | Spain (Catalonia) | clinical | 2005–2016 | 49 | 36,316 | 72% | ≥65: 83% | thresholds | 124; ≥125 | early revision, mortality, revision | lower volume |
| Arroyo 2018 [15] | none | USA (CA, FL, NY, MD) | admin. | 2007–2014 | 752 | 739,857 | 63% | mean ± SD: 67 ± 10 | hospital quartiles | 145; 267; 487; ≥488 | readmission, infection | higher volume |
| Badawy 2013 [18] | none | Norway | clinical | 1994–2010 | 54 | 26,698 | 68% | mean: 71 | thresholds | 24; 49; 99; 149; ≥150 | revision | higher volume |
| Badawy 2017 [17] | none | Norway | clinical | 2005–2015 | *67* | 28,262 | 64% | median (range): 70 (22-101) | thresholds | 49; 99; 149; ≥150 | infection | n.r. |
| Bini 2015 [32] | n.r. | USA (CA, NWR, HI) | clinical | 2009–2011 | 38 | 23,655 | 63% | mean ± SD: 68 ± 9 | thresholds | 99; 200; ≥201 | readmission | no evidence for a difference |
| Blum 2013 [34] | mixed^§^ | USA (PA) | admin. | 2001–2002 | 170 | 17,385 | 65% | ≥65: 65% | thresholds | 100; ≥100 | revision | n.r. |
| Bohm 2016 [35] | non-profit | Canada | clinical | 2006–2013 | n.r. | 238,373 | 61% | median: 68 (unilateral) | hospital quartiles | *2162; 7639; 17602; ≥17603* | mortality, infection, AE | results are inconsistent |
| Bottle 2018 [37] | n.r. | UK (England) | admin. | 2010–2015 | 138 | 311,033 | 58% | ≥70: 50% | thresholds | 25; 50; 100; 200; ≥200 | readmission, surgical compl. | n.r. |
| Charpentier 2018 [46] | none | USA (MI) | clinical | 2012–2017 | 61 | 46,709 | 58% | mean ± SD: 65 ± 12 | thresholds | 199; ≥200 | readmission | no evidence for a difference |
| Cheng 2011 [49] | n.r. | Taiwan | admin. | 2000–2003 | n.r. | 32,200 | 74% | mean: 70 | – (curve) | – | mortality, infection | higher volume |
| Cram 2011 [55] | none | USA | admin. | 2003–2005 | 2 213 | 517,867 | 66% | mean: 75 | median split by hospitals | 56; ≥57 | readmission | higher volume |
| Cram 2012 [56] | non-profit | USA | admin. | 2007–2010 | n.r. | 3,590,414 | 65% | mean: 74 | quartiles | 19; 48; 101; ≥102 | readmission | n.r. |
| Dailey 2009 [62] | n.r. | Australia (WA) | clinical | 2005–2007 | 9 | 4,858^‡^ | n.r. | n.r. | – (curve) | – | infection | higher volume |
| D'Apuzzo 2017 [60] | non-profit | USA (NY) | admin. | 1997–2014 | n.r. | 377,705 | 66% | mean: 67 | thresholds | 89; 235; 644; ≥645 | readmission | higher volume |
| Day 2019 [64] | n.r. | USA (NY) | admin. | 2009–2011 | n.r. | *94,326* | n.r. | n.r. | hospital quintiles | 9; 99; 249; 499; ≥500 | readmission | no evidence for a difference |
| Dy 2014 [71] | non-profit | USA (CA, NY) | admin. | 1997–2005 | n.r. | 301,955 | 36% | median (IQR): 69 (61-76) | thresholds | 199; 400; ≥401 | revision | n.r. |
| Featherall 2019 [79] | n.r. | USA (*OH*) | clinical | 2013–2015 | 11 | 6,760 | 62% | mean: 66 | thresholds | 100; ≥101 | AE, LOS, satisfaction | n.r. |
| Feinglass 2004 [80] | non-profit | USA (IL) | admin. | 1993–1999 | 65 | 35,531 | 66% | mean ± SD: 69 ± 10 | patient quintiles | 50; 85; 120; 180; ≥181 | AE, LOS | no evidence for a difference |
| Fry 2017 [86] | none | USA | admin. | 2010–2012 | 2,349 | 672,515 | n.r. | n.r. | patient deciles | *67; 89; 118; 148; 193; 247; 315; 415; 611; ≥612* | AE | higher volume |
| Grouven 2008 [93, 112, 214] | n.r. | Germany | admin. | 2004 | 1,016 | 110,349 | 70% | mean ± SD: 70 ± 8 | quartiles^*^ | 112; 341; ≥342 | infection, function | higher volume |
| Gutierrez 1998 [94] | non-profit | USA | admin. | 1989 | 2,916 | 67,041 | 68% | mean: 74 | thresholds | 12; 24; 50; 75; 100; ≥101 | mortality, infection | higher volume |
| Heck 1998 [98] | non-profit | USA (IN) | clinical | 1992–1993 | 25 | 291 | 63% | mean ± SD: 70 ± 0.4 | thresholds | 49; ≥50 | AE, function | higher volume |
| Hentschker 2018 [100, 101] | non-profit | Germany | admin. | 2005–2007 | 1 025 (mean) | 377,744 | 69% | mean: 70 | patient tertiles | n.r. | mortality | higher volume |
| Hervey 2003 [102] | mixed^†^ | USA | admin. | 1997 | n.r. | 55,510 | n.r. | n.r. | thresholds | 84; 149; 249; ≥250 | mortality, infection, AE, LOS | higher volume |
| Husted 2006 [110] | n.r. | Denmark | admin. | 2004 | 41 | 4,150 | n.r. | n.r. | thresholds | 49; 99; ≥100 | mortality, readmission, LOS, surgical compl. | no evidence for a difference |
| Jeschke 2017 [121] | n.r. | Germany | admin. | 2012 | 966 | 45,165 | 68% | ≥70: 59% | hospital quintiles | 56; 93; 144; 251; ≥252 | early revision, revision | higher volume |
| Judge 2006 [124] | non-profit | UK (England) | admin. | 1997–2002 | unknown | *205,321* | *59%* | n.r. | thresholds | 50; 100; 250; 500; ≥501 | mortality, revision, readmission, LOS | higher volume |
| Katz 2004 [126, 127] | non-profit | USA | admin. | 2000 | 3,122 | 80,904 | 67% | >75: 41% | thresholds | 25; 100; 200; ≥201 | mortality, infection, AE | higher volume |
| Kreder 2003 [136] | n.r. | Canada (ON) | admin. | 1992–1996 | 88 | 14,352 | 62% | mean: 70 | hospital quintiles* | 47; 113; ≥114 | early revision, mortality, revision, infection, AE, LOS | higher volume |
| Lee 2016 [146] | n.r. | China (Hong Kong) | clinical | 2011–2014 | 15 | 6,588 | n.r. | mean ± SD: 69 ± 9 | n.r. | n.r. | mortality | no evidence for a difference |
| Lenguerrand 2019 [147] | non-profit | UK (England, Wales) | clinical | 2003–2013 | 449 | *570,235* | 68% | mean (IQR):  70 (63-76) | patient quartiles | 150; 285; 440; ≥441 | infection | lower volume |
| Maman 2019 [161] | non-profit | USA (NY, FL, MD, KY) | admin. | 2007–2014 | *827* | 922,819 | 63% | mean ± SD: 67 ± 10 | patient quartiles | *145; 267; 487; ≥488* | mortality, AE, LOS | higher volume |
| Manley 2009 [163, 188] | for-profit^$^ | USA | admin. | 1997–2004 | n.r. | 53,971 | n.r. | n.r. | thresholds | 25; 100; 200; ≥201 | revision | higher volume |
| Meehan 2014 [168] | none | USA (CA) | admin. | 2005–2009 | 300 | 120,538 | 62% | ≥65: 62% | thresholds | 49; 100; 200; ≥201 | early revision, infection | higher volume |
| Meyer 2011 [170] | non-profit | Germany | clinical | 2003–2008 | 71 | 43,180^‡^ | n.r. | n.r. | thresholds | 50; 100; ≥101 | infection | higher volume |
| Mitsuyasu 2006 [171] | none | Japan | admin. | 2001–2003 | 19 | 734 | 84% | mean: 71 | thresholds | 39; ≥40 | LOS | higher volume |
| Muilwijk 2007 [176] | n.r. | Netherlands | clinical | 1996–2003 | 29 | 6,357 | 78% | mean ± SD: 71 ± 10 | hospital tertiles | 38; 60; ≥61 | infection | no evidence for a difference |
| Namba 2013a [178] | n.r. | USA (CA, CO, GA, HI, NWR, MAR) | clinical | 2001–2010 | 48 | 64,017 | 63% | mean ± SD: 67 ± 10 | thresholds | 99; 199; ≥200 | revision | no evidence for a difference |
| Namba 2013b [179] | none | USA (6 regions) | clinical | 2001–2009 | 45 | 56,216 | 63% | mean ± SD: 67 ± 10 | thresholds | 99; 199; ≥200 | infection | lower volume |
| Nimptsch 2017a [183] | non-profit | Germany | admin. | 2009–2014 | 1,027 | 842,844 | 65% | mean: 69 | patient quintiles | 56; 125; 195; 292; ≥477 | mortality | higher volume |
| Nimptsch 2017b [184] | n.r. | Germany | admin. | 2006–2013 | 1,011 | 1,093,296 | 66% | n.r. | thresholds | 49; ≥50 | mortality, LOS | higher volume |
| Norton 1998 [185] | non-profit | USA | admin. | 1985–1990 | n.r. | 295,473 | n.r. | mean: 74 | thresholds | 20; 40; 80; ≥81 | AE | higher volume |
| Ohmann 2010 [88, 187] | non-profit | Germany | admin. | 2004–2006 | 1,025 (mean) | 354,593 | 70% | median (IQR): 71 (65-76) | thresholds | 49; 99; 199; 299; ≥300 | infection, wound compl. | higher volume |
| Pamilo 2015 [190] | n.r. | Finland | clinical | 1998–2010 | 80 | 59,696 | 69% | ≥70: 55% | thresholds | 99; 249; 449; ≥450 | revision, readmission, LOS | results are inconsistent |
| Pamilo 2018 [191] | none | Finland | clinical | 2009–2013 | 4 | *4,256* | *65%* | mean: *69* | individual hospitals | 184; 219; 251; 321 | early revision, mortality, LOS | n.r. |
| Paterson 2010 [193] | non-profit | Canada (ON) | admin. | 2000–2004 | 65 | 27,217 | 62% | ≥70: 51% | patient quartiles | 130; 180; 270; ≥271 | early revision, mortality, surgical compl., LOS | results are inconsistent |
| Piuzzi 2019 [198] | none | USA (OH) | clinical | 2016–2017 | 4 | 3,270 | 60% | mean: 66 | individual hospitals | 407; 409; 439; 614 | LOS | n.r. |
| Ravi 2014 [200] | non-profit | Canada (ON) | admin. | 2002–2009 | n.r. | 71,793 | 62% | median (IQR): 68 (61-75) | – (regression) |  | infection | n.r. |
| Schaal 2017 [211] | none | Germany (SN) | other | 2010–2011 | 41 | 856 | 65% | >70: 59% | median split by patients | 257; ≥258 | satisfaction | no evidence for a difference |
| Schulze Raestrup 2006 [217] | n.r. | Germany (NRW) | admin. | 2002–2003 | 218 | 31,657 | n.r. | n.r. | thresholds | 49; 99; 199; 299; ≥300 | infection, wound compl., AE | higher volume |
| Shin 2015 [221] | n.r. | Korea | admin. | 2007–2012 | n.r. | 260,068 | 88% | mean ± SD: 69 ± 7 | thresholds | 19; 199; ≥200 | revision | higher volume |
| Singh 2011 [224] | non-profit | USA (PA) | admin. | 2001–2002 | 169 | 19,418 | 65% | mean (IQR): 69 (60-75) | thresholds | 25; 100; 200; ≥201 | mortality, infection, AE | higher volume |
| Solomon 2006 [228] | non-profit | USA (IL, NC, OH, TN) | admin. | 2000 | 276 | 9,073 | 69% | mean ± SD: 74 ± 6 | thresholds | 22; ≥23 | AE | higher volume |
| Song 2012 [229] | non-profit | Korea | clinical | 2006–2009 | 25 | 3,426 | 89% | mean: 69 | thresholds | 120; ≥121 | infection | higher volume |
| Soohoo 2006 [231, 233, 234] | none | USA (CA) | admin. | 1991–2001 | 413 | 222,684 | 62% | mean ± SD: 69 ± 10 | hospital quintiles* | means: 13; 50; 145 | mortality, readmission, infection, AE | higher volume |
| Street 2014 [237] | non-profit | UK (England) | admin. | 2009–2010 | 143 | 17,444 | 55% | mean ± SD: 69 ± 9 | – (regression) | – | LOS, function, QoL | n.r. |
| Styron 2011 [238] | n.r. | USA (35 HCUP states) | admin. | 2002 | *688* | 67,713 | 65% | median (IQR): 68 (60-75) | thresholds | 100; 197; 293; ≥294 | LOS | higher volume |
| Taylor 1997 [240] | n.r. | USA | admin. | 1992–1994 | n.r. | 276,362 | 65% | mean ± SD: 73 ± 5 | thresholds | 24; 199; ≥200 | mortality | higher volume |
| Tsai 2018 [243] | non-profit | Taiwan | admin. | 2002–2012 | n.r. | 12,843 | 76% | ≥65: 50% | hospital quartiles* | n.r. | revision, infection | no evidence for a difference |
| Varagunam 2015 [244] | n.r. | UK (England) | other | 2009–2012 | 230 | 83,648 | 57% | mean ± SD: 69 ± 9 | thresholds | 50; 100; 250; 500; ≥501 | function, QoL | no evidence for a difference |
| Wei 2010 [251] | none | Taiwan | admin. | 2000–2003 | 295 | 31,618 | 74% | mean: 74 | hospital quartiles* | 6; 23; ≥24 | infection, AE, LOS | n.r. |
| Welsh 2017 [252] | non-profit | USA | admin. | 2009–2011 | 2,500 | 608,031 | 64% | mean ± SD: 74 ± 6 | hospital quartiles | 19; 58; 137; ≥138 | readmission | n.r. |
| Wilson 2016 [254] | none | USA (NY) | admin. | 1997–2011 | n.r. | 289,976 | 62% | mean ± SD: 67 ± 11 | thresholds | 89; 235; 644; ≥645 | mortality, AE | higher volume |
| Yasunaga 2009 [256] | n.r. | Japan | other | 2005 | 345 | 3,577 | 83% | mean ± SD: 73 ± 8 | thresholds | 9; 29; 49; 99; ≥100 | AE, LOS | results are inconsistent |
| Yu 2019 [257] | non-profit | Taiwan | admin. | 2007–2008 | 437 | 30,828 | 75% | mean ± SD: 70 ± 8 | thresholds | 74; ≥75 | readmission | no evidence for a difference |

All studies were cohort studies. For studies with more than one publication, data are presented for the study which contributed most to the results. Unpublished data provided by study authors in italic. admin. = administrative; AE = postoperative adverse events; CA = California; CO = Colorado; coll. = collection; compl. = complications; FL = Florida; GA = Georgia; HCUP = Health Care Utilization Project; HI = Hawaii; IL = Illinois; IN = Indiana; KY = Kentucky; LOS = length of stay; MAR = Mid-Atlantic region; MD = Maryland; MI = Michigan; n.r. = not reported; NC = North Carolina; NRW = North-Rhine Westphalia; NWR = North-West region; NY = New York State; OH = Ohio; ON = Ontario; PA = Pennsylvania; QoL = quality of life; SN = Saxony; TN = Tennessee; UK = United Kingdom; USA = United States of America; WA = Western Australia. ^§^ includes funding by Zimmer, Smith & Nephew (medical devices co.); ^†^ includes funding by Bristol-Meyers Squibb (pharmaceutical co.); ^$^ Stryker Orthopaedics, Inc. (medical devices co.); ^‡^ number of TKAs (number of patients not reported); ^*^ some quantiles were combined.

# Supplementary Table 10. Risk of bias assessment by outcome and data

| **Study** | **Publication year** | | **Bias due to confounding** | | **Bias in selection of participants into the study** | **Bias in classification of interventions** | **Bias due to deviations from intended interventions** | | **Bias due to missing data** | | **Bias in measurement of outcomes** | | **Bias in selection of the reported result** | | **Overall risk of bias** |
| --- | --- | --- | --- | --- | --- | --- | --- | --- | --- | --- | --- | --- | --- | --- | --- |
| **Early revision ≤12 months; adjusted data** | | | | | | | | | | | | | | | |
| Kreder[136] | 2003 | | Serious | | Low | Low | Low | | Low | | Low | | Moderate | | Serious |
| Manley[163] | 2009 | | Serious | | Low | Moderate | Low | | Low | | Low | | Moderate | | Serious |
| Pamilo[191] | 2018 | | Serious | | Low | Low | Low | | Low | | Serious | | Moderate | | Serious |
| Paterson[193] | 2010 | | Serious | | Low | Low | Low | | Low | | Low | | Moderate | | Serious |
| **Early revision ≤12 months; unadjusted data** | | | | | | | | | | | | | | | |
| Arias-de la Torre[13] | 2019 | | Moderate | | Low | Low | Low | | Moderate | | Low | | Moderate | | Moderate |
| Jeschke[121] | 2017 | | Serious | | Low | Low | Low | | Low | | Low | | Moderate | | Serious |
| Kreder[136] | 2003 | | Serious | | Low | Low | Low | | Low | | Low | | Moderate | | Serious |
| Meehan[168] | 2014 | | Serious | | Low | Low | Low | | Low | | Low | | Moderate | | Serious |
| Paterson[193] | 2011 | | Serious | | Low | Low | Low | | Low | | Low | | Moderate | | Serious |
| **Revision ≤5 years; adjusted data** | | | | | | | | | | | | | | | |
| Arias-de la Torre[13] | 2019 | | Moderate | | Low | Low | Low | | Serious | | Low | | Moderate | | Serious |
| Jeschke[121] | 2017 | | Moderate | | Low | Low | Low | | Low | | Low | | Moderate | | Moderate |
| Judge[124] | 2006 | | Serious | | Low | Low | Low | | Serious | | Low | | Moderate | | Serious |
| Kreder [136] | 2003 | | Serious | | Low | Low | Low | | NI | | Low | | Moderate | | Serious |
| Manley[163] | 2009 | | Serious | | Low | Moderate | Low | | NI | | Low | | Moderate | | Serious |
| Namba[178] | 2013 | | Serious | | Low | Low | Low | | Low | | Low | | Moderate | | Serious |
| **Revision ≤5 years; unadjusted data** | | | | | | | | | | | | | | | |
| Arias-de la Torre[13] | 2019 | | Moderate | | Low | Low | Low | | Serious | | Low | | Moderate | | Serious |
| Jeschke[121] | 2017 | | Serious | | Low | Low | Low | | Low | | Low | | Moderate | | Serious |
| Judge[124] | 2006 | | Serious | | Low | Low | Low | | Serious | | Low | | Moderate | | Serious |
| Kreder[136] | 2003 | | Serious | | Low | Low | Low | | NI | | Low | | Moderate | | Serious |
| Namba[124] | 2013 | | Serious | | Low | Low | Low | | Low | | Low | | Moderate | | Serious |
| **Revision >5 years; adjusted data** | | | | | | | | | | | | | | | |
| Arias-de la Torre[13] | 2019 | | Moderate | | Low | Low | Low | | Serious | | Low | | Moderate | | Serious |
| Badawy[18] | 2013 | | Serious | | Low | Low | Low | | Low | | Low | | Moderate | | Serious |
| Dy[71] | 2014 | | Serious | | Low | Low | Low | | Low | | Low | | Moderate | | Serious |
| Pamilo[190] | 2015 | | Serious | | Low | Low | Low | | NI | | Low | | Moderate | | Serious |
| Tsai[243] | 2018 | | Serious | | Low | Low | Low | | Low | | Low | | Moderate | | Serious |
| **Revision >5 years; unadjusted data** | | | | | | | | | | | | | | | |
| Arias-de la Torre[13] | 2019 | | Moderate | | Low | Low | Low | | Serious | | Low | | Moderate | | Serious |
| Badawy[18] | 2013 | | Serious | | Low | Low | Low | | Low | | Low | | Moderate | | Serious |
| Pamilo[190] | 2015 | | Critical | | Low | Low | Low | | NI | | Low | | Moderate | | Critical |
| Shin[221] | 2015 | | Serious | | Low | Low | Low | | Low | | Low | | Moderate | | Serious |
| Tsai[243] | 2018 | | Serious | | Low | Low | Low | | Low | | Low | | Moderate | | Serious |
| **Mortality ≤3 months; adjusted data** | | | | | | | | | | | | | | | |
| Bohm[35] | 2016 | | Moderate | | Low | Low | Low | | Low | | Low | | Moderate | | Moderate |
| Hentschker[101] | 2018 | | Moderate | | Low | Low | Low | | Moderate | | Low | | Moderate | | Moderate |
| Hervey[102] | 2003 | | Serious | | Low | Low | Low | | Low | | Low | | Moderate | | Serious |
| Judge[124] | 2006 | | Moderate | | Low | Low | Low | | Low | | Low | | Moderate | | Moderate |
| Katz[126] | 2004 | | Serious | | Low | Moderate | Low | | Low | | Low | | Moderate | | Serious |
| Kreder[136] | 2003 | | Serious | | Low | Low | Low | | Low | | Low | | Moderate | | Serious |
| Maman[161] | 2019 | | Moderate | | Low | Low | Low | | Moderate | | Low | | Moderate | | Moderate |
| Nimptsch[184] | 2017 | | Moderate | | Low | Moderate | Low | | Low | | Low | | Moderate | | Moderate |
| Paterson[193] | 2010 | | Serious | | Low | Low | Low | | Low | | Low | | Moderate | | Serious |
| Singh[224] | 2011 | | Serious | | Low | Low | Low | | Low | | Low | | Moderate | | Serious |
| Soohoo[234] | 2006 | | Moderate | | Low | Low | Low | | Low | | Low | | Moderate | | Moderate |
| Wilson[254] | 2016 | | Moderate | | Low | Low | Low | | Low | | Low | | Moderate | | Moderate |
| **Mortality ≤3 months; unadjusted data** | | | | | | | | | | | | | | | |
| Arias-de la Torre[14] | 2019 | | Serious | | Low | Low | Low | | Low | | Low | | Moderate | | Serious |
| Gutierrez[94] | 1998 | | Serious | | Low | Moderate | Low | | Low | | Low | | Moderate | | Serious |
| Hervey[102] | 2003 | | Serious | | Low | Low | Low | | Low | | Low | | Moderate | | Serious |
| Husted[110] | 2006 | | Serious | | Low | Low | Low | | Low | | Low | | Moderate | | Serious |
| Judge[124] | 2006 | | Serious | | Low | Low | Low | | Low | | Low | | Moderate | | Serious |
| Katz[126] | 2004 | | Serious | | Low | Moderate | Low | | Low | | Low | | Moderate | | Serious |
| Kreder[136] | 2003 | | Serious | | Low | Low | Low | | Low | | Low | | Moderate | | Serious |
| Lee[146] | 2016 | | Serious | | Low | Serious | Low | | NI | | Low | | Moderate | | Serious |
| Nimptsch[184] | 2017 | | Serious | | Low | Moderate | Low | | Low | | Low | | Moderate | | Serious |
| Paterson[193] | 2010 | | Serious | | Low | Low | Low | | Low | | Low | | Moderate | | Serious |
| Singh[224] | 2011 | | Serious | | Low | Low | Low | | Low | | Low | | Moderate | | Serious |
| Soohoo[234] | 2006 | | Serious | | Low | Low | Low | | Low | | Low | | Moderate | | Serious |
| Taylor[240] | 1997 | | Serious | | Low | Serious | Low | | Low | | Low | | Moderate | | Serious |
| Wilson[254] | 2016 | | Serious | | Low | Low | Low | | Low | | Low | | Moderate | | Serious |
| **Mortality >3 months; adjusted data** | | | | | | | | | | | | | | | |
| Singh[224] | 2011 | | Serious | | Low | Low | Low | | Low | | Low | | Moderate | | Serious |
| Soohoo[234] | 2006 | | Moderate | | Low | Low | Low | | Low | | Low | | Moderate | | Moderate |
| **Mortality >3 months; unadjusted data** | | | | | | | | | | | | | | | |
| Arias-de la Torre[14] | 2019 | | Serious | | Low | Low | Low | | Serious | | Low | | Moderate | | Serious |
| Cheng[49] | 2011 | | Serious | | Low | Low | Low | | NI | | Low | | Moderate | | Serious |
| Gutierrez[94] | 1998 | | Serious | | Low | Moderate | Low | | Low | | Low | | Moderate | | Serious |
| Lee[146] | 2016 | | Serious | | Low | Serious | Low | | NI | | Low | | Moderate | | Serious |
| Singh[224] | 2011 | | Serious | | Low | Low | Low | | Low | | Low | | Moderate | | Serious |
| **Readmission ≤3 months; adjusted data** | | | | | | | | | | | | | | | |
| Arroyo[15] | 2018 | | Serious | | Moderate | Low | Low | | Moderate | | Low | | Moderate | | Serious |
| Bini[32] | 2015 | | Serious | | Moderate | Low | Low | | Low | | Low | | Moderate | | Serious |
| Charpentier[46] | 2018 | | Serious | | Moderate | Low | Low | | Low | | Low | | Moderate | | Serious |
| Cram[56] | 2012 | | Moderate | | Low | Serious | Low | | Low | | Low | | Moderate | | Serious |
| D'Apuzzo[60] | 2017 | | Serious | | Low | Low | Low | | Moderate | | Low | | Moderate | | Serious |
| Featherall[79] | 2019 | | Serious | | Low | Low | Low | | Low | | Low | | Moderate | | Serious |
| Pamilo[190] | 2015 | | Serious | | Low | Low | Low | | Low | | Low | | Moderate | | Serious |
| Welsh[252] | 2017 | | Serious | | Serious | Serious | Low | | Moderate | | Low | | Moderate | | Serious |
| Yu[257] | 2019 | | Moderate | | Low | Low | Low | | Moderate | | Low | | Moderate | | Moderate |
| **Infections (deep) ≤3 months; adjusted data** | | | | | | | | | | | | | | | |
| Katz[126] | | 2004 | Serious | Low | | Moderate | | Low | | Low | | Low | | Moderate | Serious |
| Lenguerrand [147] | | 2019 | Moderate | Low | | Low | | Low | | Moderate | | Low | | Moderate | Moderate |
| **Infection (deep) ≤3 months; unadjusted data** | | | | | | | | | | | | | | | |
| Katz[126] | | 2004 | Serious | Low | | Moderate | | Low | | Low | | Low | | Moderate | Serious |
| Lenguerrand [147] | | 2019 | Serious | Low | | Low | | Low | | Low | | Low | | Moderate | Serious |
| **Infection (deep) >3 months; adjusted data** | | | | | | | | | | | | | | | |
| Anis[12] | | 2019 | Serious | Low | | Low | | Low | | Moderate | | Low | | Moderate | Serious |
| Badawy[17] | | 2017 | Serious | Low | | Low | | Low | | Moderate | | Low | | Moderate | Serious |
| Lenguerrand [147] | | 2019 | Serious | Low | | Low | | Low | | Moderate | | Low | | Moderate | Serious |
| Meehan[168] | | 2014 | Moderate | Low | | Low | | Low | | Low | | Low | | Moderate | Moderate |
| Namba[179] | | 2013 | Serious | Low | | Low | | Low | | Low | | Low | | Moderate | Serious |
| Ravi[200] | | 2014 | Serious | Low | | Low | | Low | | Moderate | | Low | | Serious | Serious |
| Song[229] | | 2012 | Serious | Low | | Low | | Low | | Low | | Low | | Moderate | Serious |
| **Infections (deep) >3 months; unadjusted data** | | | | | | | | | | | | | | | |
| Anis[12] | | 2019 | Serious | Low | | Low | | Low | | Low | | Low | | Moderate | Serious |
| Badawy[17] | | 2017 | Serious | Low | | Low | | Low | | Moderate | | Low | | Moderate | Serious |
| Dailey[62] | | 2009 | Serious | Low | | Low | | Low | | Serious | | Low | | Moderate | Serious |
| Lenguerrand [147] | | 2019 | Serious | Low | | Low | | Low | | Moderate | | Low | | Moderate | Serious |
| Namba[179] | | 2013 | Serious | Low | | Low | | Low | | Low | | Low | | Moderate | Serious |
| **Adverse events ≤3 months; adjusted data** | | | | | | | | | | | | | | | |
| Katz[126] | | 2004 | Serious | Low | | Moderate | | Low | | Low | | Low | | Moderate | Serious |
| Kreder[136] | | 2003 | Serious | Low | | Low | | Low | | Low | | Low | | Moderate | Serious |
| Maman[161] | | 2019 | Moderate | Low | | Low | | Low | | Moderate | | Low | | Moderate | Moderate |
| Norton[185] | | 1998 | Serious | Low | | Moderate | | Low | | Moderate | | Low | | Moderate | Serious |
| Schulze Raestrup[217] | | 2006 | Serious | Low | | Low | | Low | | Low | | Low | | Moderate | Serious |
| Singh[224] | | 2011 | Serious | Low | | Low | | Low | | Low | | Low | | Moderate | Serious |
| Wei[251] | | 2010 | Serious | Low | | Low | | Low | | Moderate | | Low | | Moderate | Serious |
| **Adverse events ≤3 months; unadjusted data** | | | | | | | | | | | | | | | |
| Kreder[136] | | 2003 | Serious | Low | | Low | | Low | | Low | | Low | | Moderate | Serious |
| Wei[251] | | 2010 | Serious | Low | | Low | | Low | | Moderate | | Low | | Moderate | Serious |
| **Readmission ≤3 months; unadjusted data** | | | | | | | | | | | | | | | |
| Amato[9] | 2017 | | Serious | | Low | Low | Low | | Serious | | Low | | Moderate | | Serious |
| Arroyo[15] | 2018 | | Serious | | Moderate | Low | Low | | Moderate | | Low | | Moderate | | Serious |
| Bini[32] | 2015 | | Serious | | Moderate | Low | Low | | Low | | Low | | Moderate | | Serious |
| Bottle[37] | 2018 | | Serious | | Moderate | Low | Low | | Moderate | | Low | | Moderate | | Serious |
| Cram[55] | 2011 | | Serious | | Low | Low | Low | | Low | | Low | | Moderate | | Serious |
| Day[64] | 2019 | | Serious | | Low | Low | Low | | NI | | Low | | Moderate | | Serious |
| Husted[110] | 2006 | | Serious | | Low | Low | Low | | Low | | Low | | Moderate | | Serious |
| Pamilo[190] | 2015 | | Serious | | Low | Low | Low | | Low | | Low | | Moderate | | Serious |
| Soohoo[234] | 2006 | | Serious | | Low | Low | Low | | Low | | Low | | Moderate | | Serious |
| Welsh[252] | 2017 | | Serious | | Serious | Serious | Low | | Moderate | | Low | | Moderate | | Serious |
| Yu[257] | 2019 | | Serious | | Low | Low | Low | | Moderate | | Low | | Moderate | | Serious |
| **Readmission >3 months; adjusted data** | | | | | | | | | | | | | | | |
| Judge[124] | 2006 | | Serious | | Low | Low | Low | | Serious | | Low | | Moderate | | Serious |
| **Readmission >3 months; unadjusted data** | | | | | | | | | | | | | | | |
| Judge[124] | 2006 | | Serious | | Low | Low | Low | | Serious | | Low | | Moderate | | Serious |
| **Infection (any) ≤3 months; adjusted data** | | | | | | | | | | | | | | | |
| Bohm[35] | 2016 | | Moderate | | Low | Low | Low | | Moderate | | Low | | Moderate | | Moderate |
| Hervey[102] | 2003 | | Serious | | Low | Low | Low | | Low | | Low | | Moderate | | Serious |
| Singh[224] | 2011 | | Serious | | Low | Low | Low | | Low | | Low | | Moderate | | Serious |
| Soohoo[234] | 2006 | | Moderate | | Low | Low | Low | | Low | | Low | | Moderate | | Moderate |
| Wei[251] | 2010 | | Serious | | Low | Low | Low | | Moderate | | Low | | Moderate | | Serious |
| **Infection (any) ≤3 months; unadjusted data** | | | | | | | | | | | | | | | |
| Grouven[93] | 2008 | | Serious | | Low | Low | Low | | NI | | Low | | Moderate | | Serious |
| Hervey[102] | 2003 | | Serious | | Low | Low | Low | | Low | | Low | | Moderate | | Serious |
| Singh[224] | 2011 | | Serious | | Low | Low | Low | | Low | | Low | | Moderate | | Serious |
| Soohoo[234] | 2006 | | Serious | | Low | Low | Low | | Low | | Low | | Moderate | | Serious |
| Wei[251] | 2010 | | Serious | | Low | Low | Low | | Moderate | | Low | | Moderate | | Serious |
| **Infection (any) >3 months; adjusted data** | | | | | | | | | | | | | | | |
| Kreder[136] | 2003 | | Serious | | Low | Low | Low | | Low | | Low | | Moderate | | Serious |
| Soohoo[234] | 2006 | | Moderate | | Low | Low | Low | | Low | | Low | | Moderate | | Moderate |
| Tsai[243] | 2018 | | Serious | | Low | Low | Low | | Low | | Low | | Moderate | | Serious |
| **Infection (any) >3 months; unadjusted data** | | | | | | | | | | | | | | | |
| Cheng[49] | 2011 | | Serious | | Low | Low | Low | | NI | | Low | | Moderate | | Serious |
| Kreder[136] | 2003 | | Serious | | Low | Low | Low | | Low | | Low | | Moderate | | Serious |
| Tsai[243] | 2018 | | Serious | | Low | Low | Low | | Low | | Low | | Moderate | | Serious |
| **Infection (superficial) ≤3 months; adjusted data** | | | | | | | | | | | | | | | |
| Arroyo[15] | 2018 | | Serious | | Serious | Low | Low | | Moderate | | Low | | Moderate | | Serious |
| IQWIG[112] | 2005 | | Moderate | | Low | Low | Low | | Low | | Low | | Low | | Moderate |
| Meyer[170] | 2011 | | Serious | | Low | Low | Low | | Low | | Low | | Moderate | | Serious |
| Ohmann[187] | 2010 | | Moderate | | Low | Low | Low | | Low | | Low | | Moderate | | Moderate |
| **Infection (superficial) ≤3 months; unadjusted data** | | | | | | | | | | | | | | | |
| Meyer[170] | 2011 | | Serious | | Low | Low | Low | | Low | | Low | | Moderate | | Serious |
| **Infection (superficial) >3 months; adjusted data** | | | | | | | | | | | | | | | |
| Anis[12] | 2019 | | Serious | | Low | Low | Low | | Moderate | | Low | | Moderate | | Serious |
| Muilwijk[176] | 2007 | | Serious | | Low | Low | Low | | Moderate | | Low | | Moderate | | Serious |
| Song[229] | 2012 | | Serious | | Low | Low | Low | | Low | | Low | | Moderate | | Serious |
| **Infection (superficial) >3 months; unadjusted data** | | | | | | | | | | | | | | | |
| Anis[12] | 2019 | | Serious | | Low | Low | Low | | Low | | Low | | Moderate | | Serious |
| Dailey[62] | 2009 | | Serious | | Low | Low | Low | | Serious | | Low | | Moderate | | Serious |
| Muilwijk[176] | 2007 | | Serious | | Low | Low | Low | | Moderate | | Low | | Moderate | | Serious |
| Song[229] | 2012 | | Serious | | Low | Low | Low | | Low | | Low | | Moderate | | Serious |
| **Wound complications; adjusted data** | | | | | | | | | | | | | | | |
| Ohmann[187] | 2010 | | Moderate | | Low | Low | Low | | Low | | Low | | Moderate | | Moderate |
| **Surgical complications composite; adjusted data** | | | | | | | | | | | | | | | |
| Paterson[193] | 2010 | | Serious | | Low | Low | Low | | Low | | Low | | Moderate | | Serious |
| Schulze Raestrup[217] | 2006 | | Serious | | Low | Low | Low | | Low | | Low | | Moderate | | Serious |
| **Surgical complications composite; unadjusted data** | | | | | | | | | | | | | | | |
| Bottle[37] | 2018 | | Serious | | Serious | Low | Low | | Moderate | | Low | | Moderate | | Serious |
| Gutierrez[94] | 1998 | | Serious | | Low | Moderate | Low | | Low | | Low | | Moderate | | Serious |
| Husted[110] | 2006 | | Serious | | Low | Low | Low | | Low | | Low | | Moderate | | Serious |
| **Thromboembolic event ≤3 months; adjusted data** | | | | | | | | | | | | | | | |
| Bohm[35] | 2016 | | Moderate | | Low | Low | Low | | Moderate | | Low | | Moderate | | Moderate |
| Hervey[102] | 2003 | | Serious | | Low | Low | Low | | Low | | Low | | Moderate | | Serious |
| Katz[126] | 2004 | | Serious | | Low | Moderate | Low | | Low | | Low | | Moderate | | Serious |
| Singh[224] | 2011 | | Serious | | Low | Low | Low | | Low | | Low | | Moderate | | Serious |
| Soohoo[234] | 2006 | | Moderate | | Low | Low | Low | | Low | | Low | | Moderate | | Moderate |
| **Thromboembolic event ≤3 months; unadjusted data** | | | | | | | | | | | | | | | |
| Hervey[102] | 2003 | | Serious | | Low | Low | Low | | Low | | Low | | Moderate | | Serious |
| Katz[126] | 2004 | | Serious | | Low | Moderate | Low | | Low | | Low | | Moderate | | Serious |
| Kreder[136] | 2003 | | Serious | | Low | Low | Low | | Low | | Low | | Moderate | | Serious |
| Singh[224] | 2011 | | Serious | | Low | Low | Low | | Low | | Low | | Moderate | | Serious |
| Soohoo[234] | 2006 | | Serious | | Low | Low | Low | | Low | | Low | | Moderate | | Serious |
| **Thromboembolic event >3 months; adjusted data** | | | | | | | | | | | | | | | |
| Soohoo[234] | 2006 | | Moderate | | Low | Low | Low | | Low | | Low | | Moderate | | Moderate |
| **Thrombophlebitis ≤3 months; adjusted data** | | | | | | | | | | | | | | | |
| Soohoo[234] | 2006 | | Moderate | | Low | Low | Low | | Low | | Low | | Moderate | | Moderate |
| **Thrombophlebitis ≤3 months; unadjusted data** | | | | | | | | | | | | | | | |
| Soohoo[234] | 2006 | | Serious | | Low | Low | Low | | Low | | Low | | Moderate | | Serious |
| **Thrombophlebitis >3 months; adjusted data** | | | | | | | | | | | | | | | |
| Soohoo[234] | 2006 | | Moderate | | Low | Low | Low | | Low | | Low | | Moderate | | Moderate |
| **Pneumonia ≤3 months; adjusted data** | | | | | | | | | | | | | | | |
| Katz[126] | 2004 | | Serious | | Low | Moderate | Low | | Low | | Low | | Moderate | | Serious |
| **Pneumonia ≤3 months; unadjusted data** | | | | | | | | | | | | | | | |
| Katz[126] | 2004 | | Serious | | Low | Moderate | Low | | Low | | Low | | Moderate | | Serious |
| **Myocardial infarction ≤3 months; adjusted data** | | | | | | | | | | | | | | | |
| Bohm[35] | 2016 | | Moderate | | Low | Low | Low | | Moderate | | Low | | Moderate | | Moderate |
| Katz[126] | 2004 | | Serious | | Low | Moderate | Low | | Low | | Low | | Moderate | | Serious |
| Singh[224] | 2011 | | Serious | | Low | Low | Low | | Low | | Low | | Moderate | | Serious |
| **Myocardial infarction ≤3 months; unadjusted data** | | | | | | | | | | | | | | | |
| Katz[126] | 2004 | | Serious | | Low | Moderate | Low | | Low | | Low | | Moderate | | Serious |
| Singh[224] | 2011 | | Serious | | Low | Low | Low | | Low | | Low | | Moderate | | Serious |
| **‘Composite adverse events including mortality’ ≤3 months; adjusted data** | | | | | | | | | | | | | | | |
| Featherall[79] | 2019 | | Serious | | Low | Low | Low | | Low | | Low | | Moderate | | Serious |
| Feinglass[80] | 2004 | | Serious | | Low | Low | Low | | Low | | Low | | Moderate | | Serious |
| Fry[86] | 2017 | | Serious | | Low | Serious | Low | | Moderate | | Low | | Moderate | | Serious |
| Meehan[168] | 2014 | | Moderate | | Low | Low | Low | | Low | | Low | | Moderate | | Moderate |
| Solomon[228] | 2006 | | Serious | | Low | Moderate | Low | | NI | | Low | | Moderate | | Serious |
| Wilson[254] | 2016 | | Moderate | | Low | Low | Low | | Low | | Low | | Moderate | | Moderate |
| Yasunaga[256] | 2009 | | Serious | | Low | Serious | Low | | Low | | Moderate | | Serious | | Serious |
| **‘Composite adverse events including mortality’ ≤3 months; unadjusted data** | | | | | | | | | | | | | | | |
| Feinglass[80] | 2004 | | Serious | | Low | Low | Low | | Low | | Low | | Moderate | | Serious |
| Solomon[228] | 2006 | | Serious | | Low | Moderate | Low | | NI | | Low | | Moderate | | Serious |
| Wilson[254] | 2016 | | Serious | | Low | Low | Low | | Low | | Low | | Moderate | | Serious |
| Yasunaga[256] | 2009 | | Serious | | Low | Serious | Low | | Low | | Moderate | | Serious | | Serious |
| **Length of stay; adjusted data** | | | | | | | | | | | | | | | |
| Adhia[4] | 2019 | | Serious | | Moderate | Low | Low | | Low | | Low | | Moderate | | Serious |
| Featherall[79] | 2019 | | Serious | | Low | Low | Low | | Low | | Low | | Moderate | | Serious |
| Hervey[102] | 2003 | | Serious | | Low | Low | Low | | Low | | Low | | Moderate | | Serious |
| Maman[161] | 2019 | | Moderate | | Low | Low | Low | | Moderate | | Low | | Moderate | | Moderate |
| Mitsuyasu[171] | 2006 | | Serious | | Low | Low | Low | | Low | | Low | | Moderate | | Serious |
| Paterson[193] | 2010 | | Serious | | Low | Low | Low | | Low | | Low | | Moderate | | Serious |
| Street[237] | 2014 | | Moderate | | Low | Serious | Low | | NI | | Low | | Moderate | | Serious |
| Styron[238] | 2011 | | Serious | | Low | Low | Low | | Low | | Low | | Moderate | | Serious |
| Wei[251] | 2010 | | Serious | | Low | Low | Low | | Moderate | | Low | | Moderate | | Serious |
| Yasunaga[256] | 2009 | | Serious | | Low | Serious | Low | | Low | | Moderate | | Serious | | Serious |
| **Length of stay; unadjusted data** | | | | | | | | | | | | | | | |
| Feinglass[80] | 2004 | | Serious | | Low | Low | Low | | Low | | Low | | Moderate | | Serious |
| Husted[110] | 2006 | | Serious | | Low | Low | Low | | Low | | Low | | Moderate | | Serious |
| Judge[124] | 2006 | | Serious | | Low | Low | Low | | Low | | Low | | Moderate | | Serious |
| Kreder[136] | 2003 | | Serious | | Low | Low | Low | | Low | | Low | | Moderate | | Serious |
| Mitsuyasu[171] | 2006 | | Serious | | Low | Low | Low | | Low | | Low | | Moderate | | Serious |
| Nimptsch[184] | 2017 | | Serious | | Low | Moderate | Low | | Low | | Low | | Moderate | | Serious |
| Pamilo[190] | 2015 | | Serious | | Low | Low | Low | | Low | | Low | | Moderate | | Serious |
| Styron[238] | 2011 | | Serious | | Low | Low | Low | | Low | | Low | | Moderate | | Serious |
| Wei[251] | 2010 | | Serious | | Low | Low | Low | | Moderate | | Low | | Moderate | | Serious |
| Yasunaga[256] | 2009 | | Serious | | Low | Serious | Low | | Low | | Moderate | | Serious | | Serious |
| **Health-related quality of life; adjusted data** | | | | | | | | | | | | | | | |
| Varagunam[244] | 2015 | | Serious | | Low | Low | Low | | Serious | | Low | | Moderate | | Serious |
| **Function ≤3 months; adjusted data** | | | | | | | | | | | | | | | |
| IQWIG[112] | 2009 | | Serious | | Low | Low | Low | | Moderate | | Low | | Low | | Moderate |
| **Function ≤3 months; unadjusted data** | | | | | | | | | | | | | | | |
| Heck[98] | 1998 | | Serious | | Low | Serious | Low | | Low | | Low | | Moderate | | Serious |
| **Satisfaction; adjusted data** | | | | | | | | | | | | | | | |
| Featherall[79] | 2019 | | Serious | | Low | Low | Low | | Serious | | Low | | Moderate | | Critical |
| **Satisfaction; unadjusted data** | | | | | | | | | | | | | | | |
| Schaal[211] | 2017 | | Serious | | Serious | Low | Low | | Low | | Serious | | Moderate | | Critical |

# Supplementary Table 11. Results of linear dose-response meta-analysis of best-adjusted effect estimates (additional secondary outcomes)

| **Outcome (secondary)** | ***k*** | ***(n/N)***  ***[%]*** | ***I^2^*** | **Pooled *OR* [95% *CI*] for 50 TKA/year increase** | **Risk of bias (ROBINS-I)** | **References** |
| --- | --- | --- | --- | --- | --- | --- |
| Infection (any)  (≤3 months) | 4 | 1 976/ 329 280  (0.6%) | 89 % | 0.44  [0.17 - 1.15] | serious^a^ | [102, 224, 234, 251] |
| Thromboembolic event (≤3 months) | 4 | 1 974/ 376 486  (0.5%) | 62 % | 0.96  [0.86 - 1.07] | serious^a^ | [102, 126, 224, 234] |
| Infection (superficial)  (1-2 years) | 3 | 558/ 22 324  (2.5%) | 90 % | 0.82  [0.55 - 1.24] | serious^b^ | [10, 176, 229] |

Note. Statistically significant results in bold. a = Overall risk of bias was serious in all but one study in which it was moderate; b = Overall risk of bias was serious in all studies.

Abbreviations: *CI* = confidence interval; *df* =degrees of freedom; *I^2^* = index for residual heterogeneity; *k =* number of studies; n = patients with event; *N =* number of patients at risk; *OR =* odds ratio; *Q =* test statistic for residual heterogeneity; ROBINS-I = Risk Of Bias In Non-randomized Studies of Interventions tool; TKA = total knee arthroplasty.

# Supplementary Table 12. Results of linear dose-response meta-analysis of best-available effect estimates (adjusted and unadjusted)

| **Outcome** | ***k*** | ***n/N*** | ***I^2^*** | **Pooled *OR* [95% *CI*] for 50 TKA/year increase** | **References** |
| --- | --- | --- | --- | --- | --- |
| **Primary** |  |  |  |  |  |
| Early revision | 5 | 4 715/  239 232 (2,0%) | 94 % | 0.93 [0.79 - 1.11] | [13, 121, 136, 168, 193] |
| **Secondary** |  |  |  |  |  |
| Mortality  (≤3 months) | 10 | 6 100/ 2 915 358 (0,2%) | 39 % | **0.93** [0.92 - 0.94] | [102, 124, 126, 136, 161, 184, 193, 224, 234, 240] |
| Infection (deep)  (1-4 years) | 5 | 2 414/ 780 900 (0,3%) | 68 % | 0.99 [0.93 - 1.05] | [12, 17, 62, 147, 179] |
| Revision  (6-10 years) | 4 | 5 498/ 163 520 (3,3%) | 96 % | 1.02 [0.88 - 1.18] | [13, 18, 190, 221] |
| Readmission  (≤3 months) | 8 | 126 321/ 1 467 498 (8,6%) | 99 % | **0.84** [0.73 - 0.96] | [9, 15, 37, 110, 190, 234, 257] |
| Infection (superficial)  (1-2 years) | 4 | 632/ 27 187 (2,3%) | 87 % | 0.96 [0.59 - 1.56] | [12, 62, 176, 229] |
| Surgical complications  (≤3 months) | 3 | 22 521/ 342 400 (6,6%) | 59 % | 0.94 [0.85 - 1.04] | [102, 126, 136, 224, 234] |
| Thromboembolic event (≤3 months) | 5 | 2 354/ 390 838 (0,6%) | 50 % | 0.96 [0.89 - 1.02] | [37, 110, 193] |

Note. The outcomes with no additional (unadjusted) effect estimates from other studies than those in the main dose-response analysis (revision 1-5 years, infection (any)) are not listed again. Statistically significant results in bold. Abbreviations: *CI* = confidence interval; *df* = degrees of freedom; *I^2^* = index for residual heterogeneity; *k* = number of studies; *n* = patients with event; *N =* number of patients at risk; *OR* = odds ratio; *Q* = test statistic for residual heterogeneity.

# Supplementary Table 13. Sensitivity analysis 1: univariate meta-analysis of highest vs. lowest hospital volumes

| **Outcome** | ***k*** | ***N*** | ***I^2^*** | **Pooled *OR***  **[95% *CI*]** | **References** |
| --- | --- | --- | --- | --- | --- |
| Mortality  (≤3 months) | 9 | 1 500 741 | 62 % | **0.62** [0.48 - 0.79] | [102, 124, 126, 136, 161, 184, 193, 224, 234] |
| Infection (deep)  (1-4 years) | 3 | 8 674 | 54 % | 1.60 [0.91 - 2.82] | [12, 17, 179] |
| Revision  (1-5 years) | 5 | 85 059 | 92 % | 0.99 [0.65 - 1.50] | [13, 121, 124, 136, 178] |
| Readmission  (≤3 months) | 3 | 223 825 | 72 % | **0.85** [0.74 - 0.98] | [15, 190, 257] |
| Infection (any)  (3 months) | 4 | 180 650 | 0 % | **0.57** [0.44 - 0.74] | [102, 224, 234, 251] |
| Infection (superficial)  (1-2 years) | 3 | 8 674 | 90 % | 0.67 [0.24 - 1.89] | [12, 176, 229] |
| Thromboembolic events (≤3 months) | 4 | 169 335 | 50 % | 0.82 [0.50 - 1.34] | [102, 126, 224, 234] |

Note. Statistically significant results in bold. Note. Abbreviations: *CI* = confidence interval; *df* = degrees of freedom; *I^2^* = index for residual heterogeneity; *k* = number of studies; *N =* number of patients; *OR* = odds ratio; *Q* = test statistic for residual heterogeneity.

# Supplementary Table 14. Sensitivity analysis 2: linear dose-response meta-analysis of effect estimates adjusted for age, gender and comorbidity

| **Outcome** | ***k*** | ***N*** | ***I^2^*** | **Pooled *OR* [95% *CI*] for 50 TKA/year increase** | **References** |
| --- | --- | --- | --- | --- | --- |
| Mortality  (≤3 months) | 8 | 2 433 695 | 55 % | **0.90** [0.85 - 0.95] | [102, 126, 136, 161, 184, 193, 224, 234] |
| Revision  (1-5 years) | 4 | 139 675 | 97 % | 0.94 [0.83 - 1.07] | [13, 121, 136, 178] |
| Infection (any)  (≤3 months) | 3 | 297 662 | 85 % | **0.69** [0.53 - 0.89] | [102, 224, 234] |

Note. The outcomes that included the same studies as in the dose-response meta-analysis of ‘best-adjusted’ effect estimates (Infection (deep), readmission, thromboembolic events, Table xi) are not listed again. Statistically significant results in bold. Abbreviations: *CI* = confidence interval; *df* = degrees of freedom; *I^2^* = index for residual heterogeneity; *k* = number of studies; *N =* number of patients; *OR* = odds ratio; *Q* = test statistic for residual heterogeneity.

# Supplementary Table 15. Sensitivity analysis 3: linear dose-response meta-analysis of effect estimates adjusted for age, gender, comorbidity and surgeon volume

| **Outcome (secondary)** | ***k*** | ***N*** | ***I^2^*** | **Pooled *OR* [95% *CI*] for 50 TKA/year increase** | **References** |
| --- | --- | --- | --- | --- | --- |
| Mortality  (≤3 months) | 4 | 175 953 | 33 % | 0.94 [0.87 - 1.00] | [102, 126, 136, 184, 193] |

Note. Abbreviations: *CI* = confidence interval; *df* = degrees of freedom; *I^2^* = index for residual heterogeneity; *k* = number of studies; *N =* number of patients; *OR* = odds ratio; *Q* = test statistic for residual heterogeneity.

# Supplementary Table 16. Sensitivity analysis 4: linear dose-response meta-analysis of unadjusted effect estimates

| **Outcome (secondary)** | ***k*** | ***N*** | ***I^2^*** | **Pooled *OR* [95% *CI*] for 50 TKA/year increase** | **References** |
| --- | --- | --- | --- | --- | --- |
| Mortality  (≤3 months) | 9 | 2 638 996 | 62 % | **0.94** [0.93 - 0.95] | [102, 124, 126, 136, 161, 184, 193, 224, 234] |
| Infection (deep)  (1-4 years) | 3 | 97 019 | 94 % | 0.94 [0.77 - 1.15] | [10, 17, 179] |
| Revision  (1-5 years) | 5 | 163 520 | 92 % | 0.99 [0.92 - 1.07] | [13, 121, 124, 136, 178] |
| Readmission  (≤3 months) | 3 | 830 381 | 99 % | 0.93 [0.79 - 1.10] | [15, 190, 257] |
| Infection (any)  (≤3 months) | 4 | 329 280 | 16 % | **0.88** [0.84 - 0.91] | [102, 224, 234, 251] |
| Thromboembolic event (≤3 months) | 4 | 376 486 | 35 % | 0.99 [0.95 - 1.03] | [102, 126, 224, 234] |
| Infection (superficial) (1-2 years) | 3 | 22 324 | 94 % | 0.81 [0.54 - 1.22] | [12, 176, 229] |

Note. Statistically significant results in bold. Abbreviations: *CI* = confidence interval; *df* = degrees of freedom; *I^2^* = index for residual heterogeneity; *k* = number of studies; *N =* number of patients; *OR* = odds ratio; *Q* = test statistic for residual heterogeneity.

# References

1. Abblitt WP, Ascione T, Bini S, Bori G, Brekke AC, Chen AF, et al (2018) Hip and knee section, outcomes: Proceedings of international consensus on orthopedic infections. J Occup Med Toxicol 34(2):S487-S495

2. Abdelaal MS, Restrepo C, Sharkey PF (2020) Global perspectives on arthroplasty of hip and knee joints. Orthopedic Clinics of North America 51(2):169-176

3. Adelani MA, Keller MR, Barrack RL, Olsen MA (2018) The impact of hospital volume on racial differences in complications, readmissions, and emergency department visits following total joint arthroplasty. J Arthroplasty 33(2):309-315.e320

4. Adhia AH, Feinglass JM, Suleiman LI (2019) What are the risk factors for 48 or more-hour stay and nonhome discharge after total knee arthroplasty? Results from 151 illinois hospitals, 2016-2018. J Arthroplasty 35(6):1466-1473

5. Adrados M, Theobald J, Hutzler L, Bosco J (2016) The centralization of total joint arthroplasty in new york state an analysis of 168,247 cases. Bull Hosp Jt Dis (2013) 74(4):282-286

6. Ahc M (2019) Study: Total knee replacement surgery patients who live far from hospital experience better outcomes: Surprising results led one researcher to lobby for practice changes at his facility. Same-Day Surgery 43(5):N.PAG-N.PAG

7. Ali AM, Loeffler MD, Aylin P, Bottle A (2019) Predictors of 30-day readmission after total knee arthroplasty: Analysis of 566,323 procedures in the united kingdom. J Arthroplasty 34(2):242-248.e241

8. Alibhai A, Saunders D, Johnston DW, Bay K (2001) Total hip and knee replacement surgeries in alberta utilization and associated outcomes. Healthc Manage Forum 14(2):25-32

9. Amato L, Fusco D, Acampora A, Bontempi K, Rosa AC, Colais P, et al (2017) Volume and health outcomes: Evidence from systematic reviews and from evaluation of italian hospital data. Epidemiol Prev 41(5-6 (Suppl 2)):1-128

10. Anis HK, Mahmood BM, Klika AK, Mont MA, Barsoum WK, Molloy RM, et al (2020) Hospital volume and postoperative infections in total knee arthroplasty. J Arthroplasty 35(4):1079-1083

11. Anis HK, Ramanathan D, Sodhi N, Klika AK, Piuzzi NS, Mont MA, et al (2019) Postoperative infection in cementless and cemented total knee arthroplasty: A propensity score matched analysis. J Knee Surg 32(11):1058-1062

12. Anis HK, Sodhi N, Klika AK, Mont MA, Barsoum WK, Higuera CA, et al (2019) Is operative time a predictor for post-operative infection in primary total knee arthroplasty? J Arthroplasty 34(7):S331-S336

13. Arias-de la Torre J, Pons-Cabrafiga M, Valderas JM, Evans JP, Martin V, Molina AJ, et al (2019) Influence of hospital volume of procedures by year on the risk of revision of total hip and knee arthroplasties: A propensity score-matched cohort study. J Clin Med 8(5):670

14. Arias-de la Torre J, Valderas JM, Evans JP, Martin V, Molina AJ, Munoz L, et al (2019) Differences in risk of revision and mortality between total and unicompartmental knee arthroplasty. The influence of hospital volume. J Arthroplasty 34(5):865-871

15. Arroyo NS, White RS, Gaber-Baylis LK, La M, Fisher AD, Samaru M (2018) Racial/ethnic and socioeconomic disparities in total knee arthroplasty 30- and 90-day readmissions: A multi-payer and multistate analysis, 2007-2014. Popul Health Manag 22(2):175-185

16. Averett SL, Terrizzi S, Wang Y (2019) Taking the con out of pennsylvania: Did hip/knee replacement patients benefit? A retrospective analysis. Health Policy and Technology 8(4):349-355

17. Badawy M, Espehaug B, Fenstad AM, Indrekvam K, Dale H, Havelin LI, et al (2017) Patient and surgical factors affecting procedure duration and revision risk due to deep infection in primary total knee arthroplasty. BMC Musculoskelet Disord 18(1):1-9

18. Badawy M, Espehaug B, Indrekvam K, Engesaeter LB, Havelin LI, Furnes O (2013) Influence of hospital volume on revision rate after total knee arthroplasty with cement. J Bone Joint Surg Am 95(18):e131

19. Badawy M, Espehaug B, Indrekvam K, Havelin LI, Furnes O (2014) Higher revision risk for unicompartmental knee arthroplasty in low-volume hospitals. Acta Orthop 85(4):342-347

20. Badawy M, Fenstad AM, Bartz-Johannessen CA, Indrekvam K, Havelin LI, Robertsson O, et al (2017) Hospital volume and the risk of revision in oxford unicompartmental knee arthroplasty in the nordic countries -an observational study of 14,496 cases. BMC Musculoskelet Disord 18(1):388

21. Badawy M, Fenstad AM, Furnes O (2019) Primary constrained and hinged total knee arthroplasty: 2- and 5-year revision risk compared with unconstrained total knee arthroplasty: A report on 401 cases from the norwegian arthroplasty register 1994-2017. Acta Orthop 90(5):467-472

22. Baker P, Jameson S, Critchley R, Reed M, Gregg P, Deehan D (2013) Center and surgeon volume influence the revision rate following unicondylar knee replacement: An analysis of 23,400 medial cemented unicondylar knee replacements. J Bone Joint Surg Am 95(8):702-709

23. Baker PN, Deehan DJ, Lees D, Jameson S, Avery PJ, Gregg PJ, et al (2012) The effect of surgical factors on early patient-reported outcome measures (proms) following total knee replacement. J Bone Joint Surg Br 94(8):1058-1066

24. Bannister G, Ahmed M, Bannister M, Bray R, Dillon P, Eastaugh-Waring S (2010) Early complications of total hip and knee replacement: A comparison of outcomes in a regional orthopaedic hospital and two independent treatment centres. Ann R Coll Surg Engl 92(7):610-614

25. Barksfield R, Murray J, Robinson J, Porteous A (2017) Implications of the getting it right first time initiative for regional knee arthroplasty services. Knee 24(5):1191-1197

26. Barnett ML, Wilcock A, Michael McWilliams J, Epstein AM, Mehrotra A (2019) Are safety net and smaller hospitals being left behind under new payment models? Impact of medicare'smandatory bundled payment program across different hospital groups. Journal of general internal medicine 34(2):132

27. Barrett J, Baron JA, Losina E, Wright J, Mahomed NN, Katz JN (2006) Bilateral total knee replacement: Staging and pulmonary embolism. J Bone Joint Surg Am 88(10):2146-2151

28. Basilico FC, Sweeney G, Losina E, Gaydos J, Skoniecki D, Wright EA, et al (2008) Risk factors for cardiovascular complications following total joint replacement surgery. Arthritis Rheum 58(7):1915-1920

29. Berg U, Berg M, Rolfson O, Erichsen-Andersson A (2019) Fast-track program of elective joint replacement in hip and knee—patients' experiences of the clinical pathway and care process. Journal of Orthopaedic Surgery & Research 14(1):1-8

30. Bhattacharyya T, Freiberg AA, Mehta P, Katz JN, Ferris T (2009) Measuring the report card: The validity of pay-for-performance metrics in orthopedic surgery. Health Aff (Millwood) 28(2):526-532

31. Bhattacharyya T, Mehta P, Freiberg AA (2008) Hospital characteristics associated with success in a pay-for-performance program in orthopaedic surgery. J Bone Joint Surg Am 90(6):1240-1243

32. Bini SA, Inacio MCS, Cafri G (2015) Two-day length of stay is not inferior to 3 days in total knee arthroplasty with regards to 30-day readmissions. J Arthroplasty 30(5):733-738

33. Blum K, de Cruppe W, Ohmann C, Geraedts M (2008) Minimum hospital volumes for total knee replacement. Gesundheitswesen 70(4):209-218

34. Blum MA, Singh JA, Lee GC, Richardson D, Chen W, Ibrahim SA (2013) Patient race and surgical outcomes after total knee arthroplasty: An analysis of a large regional database. Arthritis Care Res (Hoboken) 65(3):414-420

35. Bohm ER, Molodianovitsh K, Dragan A, Zhu N, Webster G, Masri B, et al (2016) Outcomes of unilateral and bilateral total knee arthroplasty in 238,373 patients. Acta Orthop 87:24-30

36. Bordini B, Stea S, Falcioni S, Ancarani C, Toni A (2014) Unicompartmental knee arthroplasty: 11-year experience from 3929 implants in ripo register. Knee 21(6):1275-1279

37. Bottle A, Loeffler MD, Aylin P, Ali AM (2018) Comparison of 3 types of readmission rates for measuring hospital and surgeon performance after primary total hip and knee arthroplasty. J Arthroplasty 33(7):2014-2019.e2012

38. Bozic KJ, Maselli J, Pekow PS, Lindenauer PK, Vail TP, Auerbach AD (2010) The influence of procedure volumes and standardization of care on quality and efficiency in total joint replacement surgery. J Bone Joint Surg Am 92(16):2643-2652

39. Brennan-Olsen SL, Vogrin S, Graves S, Holloway-Kew KL, Page RS, Sajjad MA, et al (2019) Revision joint replacement surgeries of the hip and knee across geographic region and socioeconomic status in the western region of victoria: A cross-sectional multilevel analysis of registry data. BMC Musculoskelet Disord 20(1):300

40. Brodke DJ, Guo C, Aouad M, Brown TT, Bozic KJ (2019) Impact of reference pricing on cost and quality in total joint arthroplasty. J Bone Joint Surg Am 101(24):2212-2218

41. Browne JA, Cancienne JM, Casp AJ, Novicoff WM, Werner BC (2018) Certificate-of-need state laws and total knee arthroplasty. J Arthroplasty 33(7):2020-2024

42. Cai X, Cram P, Vaughan-Sarrazin M (2012) Are african american patients more likely to receive a total knee arthroplasty in a low-quality hospital? Clin Orthop Relat Res 470(4):1185-1193

43. Campbell M, McKenzie JE, Sowden A, Katikireddi SV, Brennan SE, Ellis S, et al (2020) Synthesis without meta-analysis (swim) in systematic reviews: Reporting guideline. BMJ 368:l6890

44. Cary MP, Goode V, Crego N, Thornlow D, Colón-Emeric C, van Houtven C, et al (2019) Hospital readmission and costs of total knee replacement surgery in 2009 and 2014: Potential implications for health care managers. Health Care Manager 38(1):24-28

45. Charette RS, Sloan M, DeAngelis RD, Lee GC (2019) Higher rate of early revision following primary total knee arthroplasty in patients under age 55: A cautionary tale. J Arthroplasty 34(12):2918-2924

46. Charpentier PM, Srivastava AK, Zheng H, Ostrander JD, Hughes RE (2018) Readmission rates for one versus two-midnight length of stay for primary total knee arthroplasty analysis of the michigan arthroplasty registry collaborative quality initiative (marcqi) database. J Bone Joint Surg Am 100(20):1757-1764

47. Cheah C, Hussein IH, El Othmani A, Rizvi SA, Sayeed Z, El-Othmani MM (2020) Assessing preoperative risk factors with sex disparities in total joint arthroplasty patients and financial outcomes from the national inpatient sample database. J Am Acad Orthop Surg 28(21):e969-e976

48. Chen JC, Shaw JD, Ma Y, Rhoads KF (2016) The role of the hospital and health care system characteristics in readmissions after major surgery in california. Surgery 159(2):381-388

49. Cheng CH, Cheng YT, Chen JS (2011) A learning curve of total knee arthroplasty (tka) based on surgical volume analysis. Arch Gerontol Geriatr 53(1):e5-9

50. Chhabra KR, Nuliyalu U, Dimick JB, Nathan H (2019) Who will be the costliest patients? Using recent claims to predict expensive surgical episodes. Med Care 57(11):869-874

51. Cobb AN, Erickson TR, Kothari AN, Eguia E, Brownlee SA, Yao W, et al (2018) Commercial quality “awards” are not a strong indicator of quality surgical care. Surgery (United States) 164(3):379-386

52. Cook J, MacLennan G, Murray D, Price A, Fitzpatrick R, Carr A, et al (2015) What can we learn from experience? Impact of healthcare provider effects in the total or partial knee arthroplasty trial (topkat). Trials 16(2):1

53. Courtney PM, Frisch NB, Bohl DD, Della Valle CJ (2018) Improving value in total hip and knee arthroplasty: The role of high volume hospitals. J Arthroplasty 33(1):1-5

54. Cram P, Landon BE, Matelski J, Ling V, Stukel TA, Paterson JM, et al (2018) Utilization and short-term outcomes of primary total hip and knee arthroplasty in the united states and canada: An analysis of new york and ontario administrative data. Arthritis Rheumatol 70(4):547-554

55. Cram P, Lu X, Kates SL, Li Y, Miller BJ (2011) Outliers: Hospitals with consistently lower and higher than predicted joint arthroplasty readmission rates. Geriatr Orthop Surg Rehabil 2(4):135-147

56. Cram P, Lu X, Kates SL, Singh JA, Li Y, Wolf BR (2012) Total knee arthroplasty volume, utilization, and outcomes among medicare beneficiaries, 1991-2010. JAMA 308(12):1227-1236

57. Cram P, Vaughan-Sarrazin MS, Wolf B, Katz JN, Rosenthal GE (2007) A comparison of total hip and knee replacement in specialty and general hospitals. J Bone Joint Surg Am 89(8):1675-1684

58. Critchley RJ, Baker PN, Deehan DJ (2012) Does surgical volume affect outcome after primary and revision knee arthroplasty? A systematic review of the literature. Knee 19(5):513-518

59. Culler SD, Holmes AM, Gutierrez B (1995) Expected hospital costs of knee replacement for rural residents by location of service. Med Care 33(12):1188-1209

60. D'Apuzzo M, Westrich G, Hidaka C, Jung Pan T, Lyman S (2017) All-cause versus complication-specific readmission following total knee arthroplasty. J Bone Joint Surg Am 99(13):1093-1103

61. Dahl AD, Robertsson O, Lidgren L (2010) Surgery for knee osteoarthritis in younger patients. Acta Orthop 81(2):161-164

62. Dailey L, Van Gessel H, Peterson A (2009) Two years of surgical site infection surveillance in western australia: Analysing variation between hospitals. Healthcare Infection 14(2):51-60

63. Davoli M, Amato L, Minozzi S, Bargagli AM, Vecchi S, Perucci CA (2005) [volume and health outcomes: An overview of systematic reviews]. Epidemiol Prev 29(3-4 Suppl):3-63

64. Day MS, Karia R, Hutzler L, Bosco JA (2019) Higher hospital costs do not result in lower readmission rates following total joint arthroplasty. Bulletin of the Hospital for Joint Diseases 77(2):136-139

65. de Cruppe W, Geraedts M (2016) [how steady are hospitals in complying with minimum volume standards? A retrospective longitudinal data analysis of the years 2006, 2008, and 2010]. Zentralbl Chir 141(4):425-432

66. de Cruppe W, Malik M, Geraedts M (2014) Achieving minimum caseload requirements: An analysis of hospital quality control reports from 2004-2010. Dtsch Arztebl Int 111(33-34):549-555

67. de Cruppe W, Malik M, Geraedts M (2015) Minimum volume standards in german hospitals: Do they get along with procedure centralization? A retrospective longitudinal data analysis. BMC Health Serv Res 15:279

68. Dowsey MM, Robertsson O, Sundberg M, Lohmander LS, Choong PFM, A WD (2017) Variations in pain and function before and after total knee arthroplasty: A comparison between swedish and australian cohorts. Osteoarthritis Cartilage 25(6):885-891

69. Duffy GP (2011) Maximizing surgeon and hospital total knee arthroplasty volume using customized patient instrumentation and swing operating rooms. Am J Orthop (Belle Mead NJ) 40(11 Suppl):5-8

70. Dy CJ, Bozic KJ, Padgett DE, Pan TJ, Marx RG, Lyman S (2014) Is changing hospitals for revision total joint arthroplasty associated with more complications? Clin Orthop Relat Res 472(7):2006-2015

71. Dy CJ, Marx RG, Bozic KJ, Pan TJ, Padgett DE, Lyman S (2014) Risk factors for revision within 10 years of total knee arthroplasty. Clin Orthop Relat Res 472(4):1198-1207

72. Dy CJ, Marx RG, Ghomrawi HM, Pan TJ, Westrich GH, Lyman S (2015) The potential influence of regionalization strategies on delivery of care for elective total joint arthroplasty. J Arthroplasty 30(1):1-6

73. Edelstein AI, Kwasny MJ, Suleiman LI, Khakhkhar RH, Moore MA, Beal MD, et al (2015) Can the american college of surgeons risk calculator predict 30-day complications after knee and hip arthroplasty? J Arthroplasty 30(9 Suppl):5-10

74. Eftekhary N, Feng JE, Anoushiravani AA, Schwarzkopf R, Vigdorchik JM, Long WJ (2019) Hospital consumer assessment of healthcare providers and systems: Do patient demographics affect outcomes in total knee arthroplasty? J Arthroplasty 34(8):1570-1574

75. El-Galaly A, Kappel A, Nielsen PT, Jensen SL (2019) Revision risk for total knee arthroplasty converted from medial unicompartmental knee arthroplasty: Comparison with primary and revision arthroplasties, based on mid-term results from the danish knee arthroplasty registry. J Bone Joint Surg Am 101(22):1999-2006

76. El-Othmani MM, Sayeed Z, Ramsey JA, Abaab L, Little BE, Saleh KJ (2019) The joint utilization management program-implementation of a bundle payment model and comparison between year 1 and 2 results. J Arthroplasty 34(11):2532-2537

77. Ellenrieder M, Lenz R, Haenle M, Bader R, Mittelmeier W (2011) Two-stage revision of implant-associated infections after total hip and knee arthroplasty. GMS Krankenhhyg Interdiszip 6(1):Doc17

78. Falbrede I, Widmer M, Kurtz S, Schneidmuller D, Dudda M, Roder C (2011) Utilization rates of lower extremity prostheses in germany and switzerland: A comparison of the years 2005-2008. Orthopade 40(9):793-801

79. Featherall J, Brigati DP, Arney AN, Faour M, Bokar DV, Murray TG, et al (2019) Effects of a total knee arthroplasty care pathway on cost, quality, and patient experience: Toward measuring the triple aim. J Arthroplasty 34(11):2561-2568

80. Feinglass J, Amir H, Taylor P, Lurie I, Manheim LM, Chang RW (2004) How safe is primary knee replacement surgery? Perioperative complication rates in northern illinois, 1993-1999. Arthritis Rheum 51(1):110-116

81. Feinglass J, Koo S, Koh J (2004) Revision total knee arthroplasty complication rates in northern illinois. Clin Orthop Relat Res (429):279-285

82. Finkelstein A, Ji Y, Mahoney N, Skinner J (2018) Mandatory medicare bundled payment program for lower extremity joint replacement and discharge to institutional postacute care: Interim analysis of the first year of a 5-year randomized trial. Jama 320(9):892‐900

83. Fontana MA, Lyman S, Islam W, MacLean CH (2019) When stars do not align: Overall hospital quality star ratings and the volume-outcome association. JB JS Open Access 4(1):e0044

84. Frisch NB, Courtney PM, Darrith B, Copeland LA, Gerlinger TL (2020) Veterans undergoing total hip and knee arthroplasty: 30-day outcomes as compared to the general population. J Am Acad Orthop Surg 28(22):923-929

85. Frisch NB, Courtney PM, Darrith B, Della Valle CJ (2017) Do higher-volume hospitals provide better value in revision hip and knee arthroplasty? Bone Joint J 99-b(12):1611-1617

86. Fry DE, Pine M, Nedza SM, Locke DG, Reband AM, Pine G (2017) Risk-adjusted hospital outcomes in medicare total joint replacement surgical procedures. J Bone Joint Surg Am 99(1):10-18

87. Garriga C, Leal J, Sanchez-Santos MT, Arden N, Price A, Prieto-Alhambra D, et al (2019) Geographical variation in outcomes of primary hip and knee replacement. JAMA Netw Open 2(10):e1914325

88. Geraedts M, Cruppe WD, Blum K, Ohmann C (2008) Implementation and effects of germany's minimum volume regulations results of the accompanying research. Dtsch Arztebl International 105(51-52):890-896

89. Gioe TJ, Killeen KK, Hoeffel DP, Bert JM, Comfort TK, Scheltema K, et al (2003) Analysis of unicompartmental knee arthroplasty in a community-based implant registry. Clin Orthop Relat Res 416:111-119

90. Goltz DE, Baumgartner BT, Politzer CS, DiLallo M, Bolognesi MP, Seyler TM (2018) The american college of surgeons national surgical quality improvement program surgical risk calculator has a role in predicting discharge to post-acute care in total joint arthroplasty. J Arthroplasty 33(1):25-29

91. Graham JE, Deutsch A, O'Connell AA, Karmarkar AM, Granger CV, Ottenbacher KJ (2013) Inpatient rehabilitation volume and functional outcomes in stroke, lower extremity fracture, and lower extremity joint replacement. Med Care 51(5):404-412

92. Graham SM, Moffat C, Lubega N, Mkandawire N, Burgess D, Harrison WJ (2018) Total knee arthroplasty in a low-income country: Short-term outcomes from a national joint registry. JB JS Open Access 3(1):e0029

93. Grouven U, Kuchenhoff H, Schrader P, Bender R (2008) Flexible regression models are useful tools to calculate and assess threshold values in the context of minimum provider volumes. J Clin Epidemiol 61(11):1125-1131

94. Gutierrez B, Culler SD, Freund DA (1998) Does hospital procedure-specific volume affect treatment costs? A national study of knee replacement surgery. Health Serv Res 33(3 Pt 1):489-511

95. Halder AM, Gehrke T, Gunster C, Heller KD, Leicht H, Malzahn J, et al (2019) Low hospital volume increases re-revision rate following aseptic revision total knee arthroplasty: An analysis of 23,644 cases. J Arthroplasty 35(4):1054-1059

96. Hamel MB, Henderson WG, Khuri SF, Daley J (2005) Surgical outcomes for patients aged 80 and older: Morbidity and mortality from major noncardiac surgery. J Am Geriatr Soc 53(3):424-429

97. Hansen EN, Ong KL, Lau E, Kurtz SM, Lonner JH (2019) Unicondylar knee arthroplasty has fewer complications but higher revision rates than total knee arthroplasty in a study of large united states databases. J Arthroplasty 34(8):1617-1625

98. Heck DA, Robinson RL, Partridge CM, Lubitz RM, Freund DA (1998) Patient outcomes after knee replacement. Clin Orthop Relat Res 356:93-110

99. Hengelbrock J, Höhle M (2019) Evaluating quality of hospital care using time-to-event endpoints based on patient follow-up data. Health Services and Outcomes Research Methodology 19(4):197-214

100. Hentschker C, Mennicken R, Reifferscheid A, Thomas D, Wasem J, Wübker A (2016) Der kausale zusammenhang zwischen zahl der fälle und behandlungsqualität in der krankenhausversorgung (rwi materialien heft 101). Rheinisch-Westfälisches Institut für Wirtschaftsforschung, Essen (Germany). <http://www.rwi-essen.de/publikationen/rwi-materialien/377/>. Accessed 07 Apr 2020

101. Hentschker C, Mennicken R, Reifferscheid A, Wasem J, Wubker A (2018) Volume-outcome relationship and minimum volume regulations in the german hospital sector - evidence from nationwide administrative hospital data for the years 2005-2007. Health Econ Rev 8(1):1-14

102. Hervey SL, Purves HR, Guller U, Toth AP, Vail TP, Pietrobon R (2003) Provider volume of total knee arthroplasties and patient outcomes in the hcup-nationwide inpatient sample. J Bone Joint Surg Am 85-a(9):1775-1783

103. Hofstede SN, Ceyisakar IE, Lingsma HF, Kringos DS, Marang-van de Mheen PJ (2019) Ranking hospitals: Do we gain reliability by using composite rather than individual indicators? BMJ Quality & Safety 28(2):94-102

104. Hofstede SN, van Bodegom-Vos L, Kringos DS, Steyerberg E, Marang-van de Mheen PJ (2018) Mortality, readmission and length of stay have different relationships using hospital-level versus patient-level data: An example of the ecological fallacy affecting hospital performance indicators. BMJ Qual Saf 27(6):474-483

105. Hollenbeck B, Hoffman MA, Tromanhauser SG (2019) High-volume arthroplasty centers demonstrate higher composite quality scores and enhanced value: Perspective on higher-volume hospitals performing arthroplasty from 2001 to 2011. JBJS 102(3):362-367

106. Hooper J, Israelski RH, Schwarzkopf R (2019) Total joint arthroplasty in the public hospitals of port-au-prince, haiti: Our experience. Arthroplast Today 5(3):376-379

107. Horn SR, Liu TC, Horowitz JA, Oh C, Bortz CA, Segreto FA, et al (2018) Clinical impact and economic burden of hospital-acquired conditions following common surgical procedures. Spine (03622436) 43(22):E1358-E1363

108. Hoshijima H, Wajima Z, Nagasaka H, Shiga T (2019) Association of hospital and surgeon volume with mortality following major surgical procedures: Meta-analysis of meta-analyses of observational studies. Medicine (Baltimore) 98(44):e17712

109. Hughes RE, Zheng H, Igrisan RM, Cowen ME, Markel DC, Hallstrom BR (2018) The michigan arthroplasty registry collaborative quality initiative experience: Improving the quality of care in michigan. J Bone Joint Surg Am 100(22):e143

110. Husted H, Hansen HC, Holm G, Bach-Dal C, Rud K, Andersen KL, et al (2006) Length of stay in total hip and knee arthroplasty in danmark i: Volume, morbidity, mortality and resource utilization. A national survey in orthopaedic departments in denmark. Ugeskr Laeger 168(22):2139-2143

111. Inacio MCS, Dillon MT, Miric A, Navarro RA, Paxton EW (2017) Mortality after total knee and total hip arthroplasty in a large integrated health care system. Perm J 21

112. Institut für Qualität und Wirtschaftlichkeit im Gesundheitswesen (IQWiG) (2005) Entwicklung und anwendung von modellen zur berechnung von schwellenwerten bei mindestmengen für die knie-totalendoprothese. Abschlussbericht b05/01a. Stiftung für Qualität und Wirtschaftlichkeit im Gesundheitswesen, rechtsfähige Stiftung des bürgerlichen Rechts, Cologne (Germany). <https://www.iqwig.de/download/b05-01a_abschlussbericht_entwicklung_und_anwendung_von_modellen_zur_berechnung_von_schwellenwerten_bei_mindestmengen_fuer_die_knie-totalendoprothese.pdf?rev=117386>. Accessed 17 Feb 2021

113. IntHout J, Ioannidis JP, Borm GF (2014) The hartung-knapp-sidik-jonkman method for random effects meta-analysis is straightforward and considerably outperforms the standard dersimonian-laird method. BMC Med Res Methodol 14:25

114. Iqbal F, Shafiq B, Zamir M, Noor S, Memon N, Memon N, et al (2020) Micro-organisms and risk factors associated with prosthetic joint infection following primary total knee replacement-our experience in pakistan. Int Orthop 44(2):283-289

115. Issa K, Cherian JJ, Kapadia BH, Robinson K, Bhowmik-Stoker M, Harwin SF, et al (2015) Readmission rates for cruciate-retaining total knee arthroplasty. J Knee Surg 28(3):239-242

116. Jain NB, Higgins LD, Ozumba D, Guller U, Cronin M, Pietrobon R, et al (2005) Trends in epidemiology of knee arthroplasty in the united states, 1990-2000. Arthritis Rheum 52(12):3928-3933

117. Jansson V, Grimberg A, Melsheimer O, Perka C, Steinbruck A (2019) Orthopaedic registries: The german experience. EFORT Open Rev 4(6):401-408

118. Jarvelin J, Hakkinen U, Rosenqvist G, Remes V (2012) Factors predisposing to claims and compensations for patient injuries following total hip and knee arthroplasty. Acta Orthop 83(2):190-196

119. Jasper LL, Jones CA, Mollins J, Pohar SL, Beaupre LA (2016) Risk factors for revision of total knee arthroplasty: A scoping review. BMC Musculoskelet Disord 17:182

120. Jennings JM, Kleeman-Forsthuber LT, Bolognesi MP (2019) Medial unicompartmental arthroplasty of the knee. Journal of the American Academy of Orthopaedic Surgeons 7(5):166-176

121. Jeschke E, Citak M, Gunster C, Matthias Halder A, Heller KD, Malzahn J, et al (2017) Are tkas performed in high-volume hospitals less likely to undergo revision than tkas performed in low-volume hospitals? Clin Orthop Relat Res 475(11):2669-2674

122. Jeschke E, Gehrke T, Gunster C, Hassenpflug J, Malzahn J, Niethard FU, et al (2016) Five-year survival of 20,946 unicondylar knee replacements and patient risk factors for failure: An analysis of german insurance data. J Bone Joint Surg Am 98(20):1691-1698

123. Jeschke E, Gehrke T, Gunster C, Heller KD, Malzahn J, Marx A, et al (2018) Impact of case numbers on the 5-year survival rate of unicondylar knee replacements in germany. Z Orthop Unfall 156(1):62-67

124. Judge A, Chard J, Learmonth I, Dieppe P (2006) The effects of surgical volumes and training centre status on outcomes following total joint replacement: Analysis of the hospital episode statistics for england. J Public Health (Oxf) 28(2):116-124

125. Kane RL, Saleh KJ, Wilt TJ, Bershadsky B (2005) The functional outcomes of total knee arthroplasty. J Bone Joint Surg Am 87(8):1719-1724

126. Katz JN, Barrett J, Mahomed NN, Baron JA, Wright RJ, Losina E (2004) Association between hospital and surgeon procedure volume and the outcomes of total knee replacement. J Bone Joint Surg Am 86-a(9):1909-1916

127. Katz JN, Bierbaum BE, Losina E (2008) Case mix and outcomes of total knee replacement in orthopaedic specialty hospitals. Med Care 46(5):476-480

128. Katz JN, Mahomed NN, Baron JA, Barrett JA, Fossel AH, Creel AH, et al (2007) Association of hospital and surgeon procedure volume with patient-centered outcomes of total knee replacement in a population-based cohort of patients age 65 years and older. Arthritis Rheum 56(2):568-574

129. Kazarian GS, Lawrie CM, Barrack TN, Donaldson MJ, Miller GM, Haddad FS, et al (2019) The impact of surgeon volume and training status on implant alignment in total knee arthroplasty. J Bone Joint Surg Am 101(19):1713-1723

130. Keohane D, Power F, Cullen E, O'Neill A, Masterson E (2019) High rate of tibial debonding and failure in a popular knee replacement: A cause for concern. Knee 27(2):459-468

131. Khanuja HS, Solano MA, Sterling RS, Oni JK, Chaudhry YP, Jones LC (2019) Surgeon mean operative times in total knee arthroplasty in a variety of settings in a health system. J Arthroplasty 34(11):2569-2572

132. Kim DH, Pearson-Chauhan KM, McCarthy RJ, Buvanendran A (2018) Predictive factors for developing chronic pain after total knee arthroplasty. Journal of Arthroplasty 33(11):3372-3378

133. Kim SJ, Postigo R, Koo S, Kim JH (2014) Causes of revision following oxford phase 3 unicompartmental knee arthroplasty. Knee Surg Sports Traumatol Arthrosc 22(8):1895-1901

134. Kokko MA, Abdel MP, Berry DJ, Butler RD, Van Citters DW (2019) A retrieval analysis perspective on revision for infection. Arthroplast Today 5(3):362-370

135. Kostuj T, Schulze-Raestrup U, Noack M, Buckup K, Smektala R (2011) Minimal provider volume in total knee replacement : An analysis of the external quality assurance program of north rhine-westphalia (qs-nrw). Chirurg 82(5):425-432

136. Kreder HJ, Grosso P, Williams JI, Jaglal S, Axcell T, Wal EK, et al (2003) Provider volume and other predictors of outcome after total knee arthroplasty: A population study in ontario. Can J Surg 46(1):15-22

137. Kreder HJ, Williams JI, Jaglal S, Axcell T, Stephen D (1999) A population study in the province of ontario of the complications after conversion of hip or knee arthrodesis to total joint replacement. Can J Surg 42(6):433-439

138. Kuo AC, Raghunathan K, Lartigue AM, Bryan Iii WE, Pepin MJ, Takemoto S, et al (2019) Freedom from opioids after total knee arthroplasty. J Arthroplasty 34(5):893-897

139. Kurtz SM, Lau EC, Ong KL, Adler EM, Kolisek FR, Manley MT (2017) Which clinical and patient factors influence the national economic burden of hospital readmissions after total joint arthroplasty? Clin Orthop Relat Res 475(12):2926-2937

140. Kurtz SM, Lau EC, Ong KL, Adler EM, Kolisek FR, Manley MT (2016) Which hospital and clinical factors drive 30- and 90-day readmission after tka? J Arthroplasty 31(10):2099-2107

141. Lalmohamed A, Vestergaard P, de Boer A, Leufkens HG, van Staa TP, de Vries F (2014) Changes in mortality patterns following total hip or knee arthroplasty over the past two decades: A nationwide cohort study. Arthritis Rheumatol 66(2):311-318

142. Lau RL, Perruccio AV, Gandhi R, Mahomed NN (2012) The role of surgeon volume on patient outcome in total knee arthroplasty: A systematic review of the literature. BMC Musculoskelet Disord 13(1):250

143. Laucis NC, Chowdhury M, Dasgupta A, Bhattacharyya T (2016) Trend toward high-volume hospitals and the influence on complications in knee and hip arthroplasty. J Bone Joint Surg Am 98(9):707-712

144. Lavernia CJ, Guzman JF (1995) Relationship of surgical volume to short-term mortality, morbidity, and hospital charges in arthroplasty. J Arthroplasty 10(2):133-140

145. Lee MS, Hollenbeck BK, Oerline MK, Skolarus TA, Jacobs BL, Jen R, et al (2019) Spillover effects of the hospital readmissions reduction program on radical cystectomy readmissions. Urol Pract 6(6):350-356

146. Lee QJ, Mak WP, Wong YC (2016) Mortality following primary total knee replacement in public hospitals in hong kong. Hong Kong Med J 22(3):237-241

147. Lenguerrand E, Whitehouse MR, Beswick AD, Kunutsor SK, Foguet P, Porter M, et al (2019) Risk factors associated with revision for prosthetic joint infection following knee replacement: An observational cohort study from england and wales. Lancet Infect Dis 19(6):589-600

148. Li BY, Urish KL, Jacobs BL, He C, Borza T, Qin Y, et al (2019) Inaugural readmission penalties for total hip and total knee arthroplasty procedures under the hospital readmissions reduction program. JAMA Network Open 2(11):e1916008-e1916008

149. Liddle AD, Pandit H, Judge A, Murray DW (2016) Effect of surgical caseload on revision rate following total and unicompartmental knee replacement. J Bone Joint Surg Am 98(1):1-8

150. Lin YC, Chang CH, Chang CJ, Wang YC, Hsieh PH, Chang Y (2019) Vascular injury during primary total knee arthroplasty: A nationwide study. Journal of the Formosan Medical Association 118(1):305-310

151. Lin YC, Chang CH, Chang CJ, Wang YC, Hsieh PH, Chang Y (2018) Vascular injury during primary total knee arthroplasty: A nationwide study. J Formos Med Assoc 118(1):305-310

152. Lindberg-Larsen M, Jorgensen CC, Bagger J, Schroder HM, Kehlet H (2016) Revision of infected knee arthroplasties in denmark. Acta Orthop 87(4):333-338

153. Liu J, Wilson L, Poeran J, Fiasconaro M, David HK, Yang E, et al (2019) Trends in total knee and hip arthroplasty recipients: A retrospective cohort study. Regional Anesthesia & Pain Medicine 44(9):854-859

154. Liu JH, Zingmond DS, McGory ML, SooHoo NF, Ettner SL, Brook RH, et al (2006) Disparities in the utilization of high-volume hospitals for complex surgery. Jama 296(16):1973-1980

155. Losina E, Kessler C, Wright EA, Creel AH, Barrett JA, Fossel AH, et al (2006) Geographic diversity of low-volume hospitals in total knee replacement: Implication for regionalization policies. Medical Care 44(7):637-645

156. Losina E, Walensky RP, Kessler CL, Emrani PS, Reichmann WM, Wright EA, et al (2009) Cost-effectiveness of total knee arthroplasty in the united states: Patient risk and hospital volume. Arch Intern Med 169(12):1113-1121; discussion 1121-1112

157. Losina E, Wright EA, Kessler CL, Barrett JA, Fossel AH, Creel AH, et al (2007) Neighborhoods matter: Use of hospitals with worse outcomes following total knee replacement by patients from vulnerable populations. Arch Intern Med 167(2):182-187

158. Lu X, Hagen TP, Vaughan-Sarrazin MS, Cram P (2009) The impact of physician-owned specialty orthopaedic hospitals on surgical volume and case complexity in competing hospitals. Clin Orthop Relat Res 467(10):2577-2586

159. Ma Y, Chu H, Mazumdar M (2016) Meta-analysis of proportions of rare events-a comparison of exact likelihood methods with robust variance estimation. Commun Stat Simul Comput 45(8):3036-3052

160. Makela KT, Peltola M, Sund R, Malmivaara A, Hakkinen U, Remes V (2011) Regional and hospital variance in performance of total hip and knee replacements: A national population-based study. Ann Med 43 Suppl 1:S31-38

161. Maman SR, Andreae MH, Gaber-Baylis LK, Turnbull ZA, White RS (2019) Medicaid insurance status predicts postoperative mortality after total knee arthroplasty in state inpatient databases. J Comp Eff Res 8(14):1213-1228

162. Manickam RN, Memtsoudis SG, Mu Y, Kim J, Kshirsagar AV, Bang H Excess readmission-based penalty: Is arthroplasty different from the other outcomes? J Surg Orthop Adv 27(4):286-294

163. Manley M, Ong K, Lau E, Kurtz SM (2009) Total knee arthroplasty survivorship in the united states medicare population: Effect of hospital and surgeon procedure volume. J Arthroplasty 24(7):1061-1067

164. Manning DW (2016) Volume and quality in total joint arthroplasty: A complex relationship: Commentary on an article by nicholas c. Laucis, bse, et al.: "Trend toward high-volume hospitals and the influence on complications in knee and hip arthroplasty". Journal of Bone & Joint Surgery, American Volume 98(9):e37-31-e37-32

165. Marlow NE, Barraclough B, Collier NA, Dickinson IC, Fawcett J, Graham JC, et al (2010) Centralization and the relationship between volume and outcome in knee arthroplasty procedures. ANZ J Surg 80(4):234-241

166. Martsolf GR, Barrett ML, Weiss AJ, Kandrack R, Washington R, Steiner CA, et al (2016) Impact of race/ethnicity and socioeconomic status on risk-adjusted hospital readmission rates following hip and knee arthroplasty. J Bone Joint Surg Am 98(16):1385-1391

167. Mathes T, Rombey T, Prediger B, Goossen K, Kugler C, Pieper D. Dose-response-metaanalysen für fragestellungen in der versorgungsforschung Paper presented at: 19. Deutscher Kongress für Versorgungsforschung (DKVF)2020; Berlin.

168. Meehan JP, Danielsen B, Kim SH, Jamali AA, White RH (2014) Younger age is associated with a higher risk of early periprosthetic joint infection and aseptic mechanical failure after total knee arthroplasty. J Bone Joint Surg Am 96A(7):529-535

169. Merkow RP, Ju MH, Chung JW, Hall BL, Cohen ME, Williams MV, et al (2015) Underlying reasons associated with hospital readmission following surgery in the united states. Jama 313(5):483-495

170. Meyer E, Weitzel-Kage D, Sohr D, Gastmeier P (2011) Impact of department volume on surgical site infections following arthroscopy, knee replacement or hip replacement. BMJ Qual Saf 20(12):1069-1074

171. Mitsuyasu S, Hagihara A, Horiguchi H, Nobutomo K (2006) Relationship between total arthroplasty case volume and patient outcome in an acute care payment system in japan. J Arthroplasty 21(5):656-663

172. Mizner RL, Petterson SC, Clements KE, Zeni JA, Jr., Irrgang JJ, Snyder-Mackler L (2011) Measuring functional improvement after total knee arthroplasty requires both performance-based and patient-report assessments: A longitudinal analysis of outcomes. J Arthroplasty 26(5):728-737

173. Mohammad HR, Matharu GS, Judge A, Murray DW (2020) The effect of surgeon caseload on the relative revision rate of cemented and cementless unicompartmental knee replacements: An analysis from the national joint registry for england, wales, northern ireland and the isle of man. JBJS 102(8):644-653

174. Morris AJ, Roberts SA, Grae N, Frampton CM (2020) Surgical site infection rate is higher following hip and knee arthroplasty when cefazolin is underdosed. Am J Health Syst Pharm;10.1093/ajhp/zxz344

175. Mufarrih SH, Ghani MOA, Martins RS, Qureshi NQ, Mufarrih SA, Malik AT, et al (2019) Effect of hospital volume on outcomes of total hip arthroplasty: A systematic review and meta-analysis. J Orthop Surg Res 14(1):468

176. Muilwijk J, van den Hof S, Wille JC (2007) Associations between surgical site infection risk and hospital operation volume and surgeon operation volume among hospitals in the dutch nosocomial infection surveillance network. Infect Control Hosp Epidemiol 28(5):557-563

177. Murad MH, Mustafa RA, Schünemann HJ, Sultan S, Santesso N (2017) Rating the certainty in evidence in the absence of a single estimate of effect. Evidence Based Medicine 22(3):85-87

178. Namba RS, Cafri G, Khatod M, Inacio MC, Brox TW, Paxton EW (2013) Risk factors for total knee arthroplasty aseptic revision. J Arthroplasty 28(8 Suppl):122-127

179. Namba RS, Inacio MC, Paxton EW (2013) Risk factors associated with deep surgical site infections after primary total knee arthroplasty: An analysis of 56,216 knees. J Bone Joint Surg Am 95(9):775-782

180. Navarro SM, Haeberle HS, Mont MA, Krebs V, Ramkumar PN (2019) Stratum-specific likelihood ratio analysis: An evidence-based and pragmatic approach to meaningful thresholds in lower extremity arthroplasty. Surg Technol Int 34:415-420

181. Navathe AS, Liao JM, Dykstra SE, Wang E, Lyon ZM, Shah Y, et al (2018) Association of hospital participation in a medicare bundled payment program with volume and case mix of lower extremity joint replacement episodes. Jama 320(9):901-910

182. Naylor JM, Hart A, Harris IA, Lewin AM (2019) Variation in rehabilitation setting after uncomplicated total knee or hip arthroplasty: A call for evidence-based guidelines. BMC Musculoskelet Disord 20(1):214

183. Nimptsch U, Mansky T (2017) Hospital volume and mortality for 25 types of inpatient treatment in german hospitals: Observational study using complete national data from 2009 to 2014. BMJ Open 7(9):19

184. Nimptsch U, Peschke D, Mansky T (2017) Minimum caseload requirements and in-hospital mortality: Observational study using nationwide hospital discharge data from 2006 to 2013. Gesundheitswesen 79(10):823-834

185. Norton EC, Garfinkel SA, McQuay LJ, Heck DA, Wright JG, Dittus R, et al (1998) The effect of hospital volume on the in-hospital complication rate in knee replacement patients. Health Serv Res 33(5 Pt 1):1191-1210

186. Ohmann C, Blum K, De Cruppé W, Geraedts M (2008) Hospital volume and outcome: Is one parameter sufficient for assessment? Chirurgische Gastroenterologie Interdisziplinar 24(4):281-286

187. Ohmann C, Verde PE, Blum K, Fischer B, de Cruppe W, Geraedts M (2010) Two short-term outcomes after instituting a national regulation regarding minimum procedural volumes for total knee replacement. J Bone Joint Surg Am 92(3):629-638

188. Ong KL, Lau E, Manley M, Kurtz SM (2008) Effect of procedure duration on total hip arthroplasty and total knee arthroplasty survivorship in the united states medicare population. J Arthroplasty 23(6):127-132

189. Pamilo KJ, Peltola M, Makela K, Hakkinen U, Paloneva J, Remes V (2013) Is hospital volume associated with length of stay, readmissions and reoperations for total hip replacement? A populationbased register analysis of 78 hospitals and 54,505 replacements. Arch Orthop Trauma Surg 133(12):1747-1755

190. Pamilo KJ, Peltola M, Paloneva J, Makela K, Hakkinen U, Remes V (2015) Hospital volume affects outcome after total knee arthroplasty. Acta Orthop 86(1):41-47

191. Pamilo KJ, Torkki P, Peltola M, Pesola M, Remes V, Paloneva J (2018) Fast-tracking for total knee replacement reduces use of institutional care without compromising quality a register-based analysis of 4 hospitals and 4,256 replacements. Acta Orthop 89(2):184-189

192. Parikh ND, Chang YH, Tapper EB, Mathur AK (2019) Outcomes of patients with cirrhosis undergoing orthopedic procedures: An analysis of the nationwide inpatient sample. J Clin Gastroenterol 53(9):e356-e361

193. Paterson JM, Williams JI, Kreder HJ, Mahomed NN, Gunraj N, Wang X, et al (2010) Provider volumes and early outcomes of primary total joint replacement in ontario. Can J Surg 53(3):175-183

194. Pathak S, Ganduglia CM, Awad SS, Chan W, Swint JM, Morgan RO (2017) What factors are associated with 90-day episode-of-care payments for younger patients with total joint arthroplasty? Clin Orthop Relat Res 475(11):2808-2818

195. Pieper D, Mathes T, Neugebauer E, Eikermann M (2013) State of evidence on the relationship between high-volume hospitals and outcomes in surgery: A systematic review of systematic reviews. J Am Coll Surg 216(5):1015-1025.e1018

196. Pine M, Fry DE, Jones BL, Meimban RJ, Pine GJ (2010) Controlling costs without compromising quality: Paying hospitals for total knee replacement. Med Care 48(10):862-868

197. Pirruccio K, Mehta S, Sheth NP (2019) The association between newly accredited orthopedic residency programs and teaching hospital complication rates in lower extremity total joint arthroplasty. J Surg Educ 77(3):690-697

198. Piuzzi NS, Strnad GJ, Ali Sakr Esa W, Barsoum WK, Bloomfield MR, Brooks PJ, et al (2019) The main predictors of length of stay after total knee arthroplasty: Patient-related or procedure-related risk factors. J Bone Joint Surg Am 101(12):1093-1101

199. Ravi B, Croxford R, Austin PC, Hollands S, Paterson JM, Bogoch E, et al (2014) Increased surgeon experience with rheumatoid arthritis reduces the risk of complications following total joint arthroplasty. Arthritis Rheumatol 66(3):488-496

200. Ravi B, Croxford R, Hollands S, Paterson JM, Bogoch E, Kreder H, et al (2014) Increased risk of complications following total joint arthroplasty in patients with rheumatoid arthritis. Arthritis & Rheumatology 66(2):254-263

201. Ricciardi BF, Liu AY, Qiu B, Myers TG, Thirukumaran CP (2019) What is the association between hospital volume and complications after revision total joint arthroplasty: A large-database study. Clin Orthop Relat Res 477(5):1221-1231

202. Ricciardi BF, Oi KK, Daines SB, Lee YY, Joseph AD, Westrich GH (2017) Patient and perioperative variables affecting 30-day readmission for surgical complications after hip and knee arthroplasties: A matched cohort study. J Arthroplasty 32(4):1074-1079

203. Robertsson O, Dunbar MJ, Knutson K, Lewold S, Lidgren L (1999) The swedish knee arthroplasty register. 25 years experience. Bull Hosp Jt Dis 58(3):133-138

204. Robin LK, Harlen DH, Richard WM, Matthew FE, Douglas SW, David RM (2013) Risk factors for all-cause hospital readmission within 30 days of hospital discharge. Journal of Clinical Outcomes Management 21(5):203-214

205. Robinson JC, Brown TT (2013) Increases in consumer cost sharing redirect patient volumes and reduce hospital prices for orthopedic surgery. Health Aff (Millwood) 32(8):1392-1397

206. Rombey T, Goossen K, Breuing J, Mathes T, Hess S, Burchard R, et al (2020) Hospital volume-outcome relationship in total knee arthroplasty: Protocol for a systematic review and non-linear dose-response meta-analysis. Syst Rev 9(1):38

207. Rosas S, Buller LT, Plate J, Higuera C, Barsoum WK, Emory C (2019) Total knee arthroplasty among medicare beneficiaries with hemophilia a and b is associated with increased complications and higher costs. J Knee Surg

208. Ross TD, Dvorani E, Saskin R, Khoshbin A, Atrey A, Ward SE (2020) Temporal trends and predictors of thirty-day readmissions and emergency department visits following total knee arthroplasty in ontario between 2003 and 2016. J Arthroplasty 35(2):364-370

209. Rusk A, Bush K, Brandt M, Smith C, Howatt A, Chow B, et al (2016) Improving surveillance for surgical site infections following total hip and knee arthroplasty using diagnosis and procedure codes in a provincial surveillance network. Infect Control Hosp Epidemiol 37(6):699-703

210. Savvidou OD (2019) Corr insights®: What is the association between hospital volume and complications after revision total joint arthroplasty: A large-database study. Clin Orthop. RelatRes 477(5):1232-1234

211. Schaal T, Schoenfelder T, Klewer J, Kugler J (2017) Effects of perceptions of care, medical advice, and hospital quality on patient satisfaction after primary total knee replacement: A cross-sectional study. PLoS One 12(6):e0178591

212. Schoenfeld A, Schoenfeld AJ (2017) Corr insights®: Have the causes of revision for total and unicompartmental knee arthroplasties changed during the past two decades? Clinical Orthopaedics & Related Research 475(7):1887-1890

213. Schrader P, Ewerbeck V (2007) [experience in orthopaedic surgery with minimum provider volumes]. Chirurg 78(11):999-1011

214. Schrader P, Grouven U, Bender R (2007) Is it possible to calculate minimum provider volumes for total knee replacement using routine data? Results of a threshold value analysis of german quality assurance data for inpatient treatment. Der Orthopade 36(6):570-576

215. Schrader P, Rath T (2005) [minimum requirements in total knee replacement. Evidence report and model calculation of the healthcare situation]. Orthopade 34(3):198, 200-194, 206-199

216. Schroer WC, Calvert GT, Diesfeld PJ, Reedy ME, LeMarr AR (2008) Effects of increased surgical volume on total knee arthroplasty complications. J Arthroplasty 23(6 Suppl 1):61-67

217. Schulze Raestrup U, Smektala R (2006) Are there relevant minimum procedure volumes in trauma and orthopedic surgery? Zentralbl Chir 131(6):483-492

218. Shervin N, Rubash HE, Katz JN (2007) Orthopaedic procedure volume and patient outcomes: A systematic literature review. Clin Orthop Relat Res 457:35-41

219. Shih T, Nicholas LH, Thumma JR, Birkmeyer JD, Dimick JB (2014) Does pay-for-performance improve surgical outcomes? An evaluation of phase 2 of the premier hospital quality incentive demonstration. Ann Surg 259(4):677-681

220. Shim J, McLernon DJ, Hamilton D, Simpson HA, Beasley M, Macfarlane GJ (2018) Development of a clinical risk score for pain and function following total knee arthroplasty: Results from the trio study. Rheumatol Adv Pract 2(2):rky021

221. Shin CH, Chang CB, Cho SH, Jeong JH, Kang SB (2015) Factors associated with the incidence of revision total knee arthroplasty in korea between 2007 and 2012: An analysis of the national claim registry. BMC Musculoskelet Disord 16(1):1-8

222. Sibanda N, Copley LP, Lewsey JD, Borroff M, Gregg P, MacGregor AJ, et al (2008) Revision rates after primary hip and knee replacement in england between 2003 and 2006. PLoS Med 5(9):e179

223. Sibley RA, Charubhumi V, Hutzler LH, Paoli AR, Bosco JA (2017) Joint replacement volume positively correlates with improved hospital performance on centers for medicare and medicaid services quality metrics. J Arthroplasty 32(5):1409-1413

224. Singh JA, Kwoh CK, Boudreau RM, Lee GC, Ibrahim SA (2011) Hospital volume and surgical outcomes after elective hip/knee arthroplasty: A risk-adjusted analysis of a large regional database. Arthritis Rheum 63(8):2531-2539

225. Singh JA, Kwoh CK, Richardson D, Chen W, Ibrahim SA (2013) Sex and surgical outcomes and mortality after primary total knee arthroplasty: A risk-adjusted analysis. Arthritis Care Res (Hoboken) 65(7):1095-1102

226. Slover JD, Tosteson AN, Bozic KJ, Rubash HE, Malchau H (2008) Impact of hospital volume on the economic value of computer navigation for total knee replacement. J Bone Joint Surg Am 90(7):1492-1500

227. Sodhi N, Mont MA (2019) Does patient experience after a total knee arthroplasty predict readmission? J Arthroplasty 34(11):2573-2579

228. Solomon DH, Chibnik LB, Losina E, Huang J, Fossel AH, Husni E, et al (2006) Development of a preliminary index that predicts adverse events after total knee replacement. Arthritis Rheum 54(5):1536-1542

229. Song KH, Kim ES, Kim YK, Jin HY, Jeong SY, Kwak YG, et al (2012) Differences in the risk factors for surgical site infection between total hip arthroplasty and total knee arthroplasty in the korean nosocomial infections surveillance system (konis). Infect Control Hosp Epidemiol 33(11):1086-1093

230. Soohoo NF, Farng E, Lieberman JR, Chambers L, Zingmond DS (2010) Factors that predict short-term complication rates after total hip arthroplasty. Clin Orthop Relat Res 468(9):2363-2371

231. SooHoo NF, Lieberman JR, Ko CY, Zingmond DS (2006) Factors predicting complication rates following total knee replacement. J Bone Joint Surg Am 88(3):480-485

232. SooHoo NF, Zingmond DS, Ko CY (2008) Disparities in the utilization of high-volume hospitals for total knee replacement. J Natl Med Assoc 100(5):559-564

233. SooHoo NF, Zingmond DS, Lieberman JR, Ko CY (2006) Optimal timeframe for reporting short-term complication rates after total knee arthroplasty. J Arthroplasty 21(5):705-711

234. Soohoo NF, Zingmond DS, Lieberman JR, Ko CY (2006) Primary total knee arthroplasty in california 1991 to 2001: Does hospital volume affect outcomes? J Arthroplasty 21(2):199-205

235. Stavrakis AI, SooHoo NF, Lieberman JR (2015) Bilateral total hip arthroplasty has similar complication rates to unilateral total hip arthroplasty. J Arthroplasty 30(7):1211-1214

236. Stengel D, Ekkernkamp A, Dettori J, Hanson B, Sturmer KM, Siebert H (2004) A rapid review of the minimum quality problems using total knee arthroplasty as an example. Where do the magical threshold values come from? Unfallchirurg 107(10):967-988

237. Street A, Gutacker N, Bojke C, Devlin N, Daidone S (2014) Health services and delivery research. (eds) In: Variations in outcome and costs among nhs providers for common surgical procedures: Econometric analyses of routinely collected data*.*10.3310/hsdr02010. NIHR Journals Library. Health Services and Delivery Research, Southampton (UK)

238. Styron JF, Koroukian SM, Klika AK, Barsoum WK (2011) Patient vs provider characteristics impacting hospital lengths of stay after total knee or hip arthroplasty. J Arthroplasty 26(8):1418-1426

239. Suchman KI, Poeran J, Huang HH, Mazumdar M, Bronson M, Galatz LM, et al (2019) Are histological examinations of arthroplasty specimens performed consistently across the country? A large database study. Clin Orthop Relat Res 477(8):1815-1824

240. Taylor HD, Dennis DA, Crane HS (1997) Relationship between mortality rates and hospital patient volume for medicare patients undergoing major orthopaedic surgery of the hip, knee, spine, and femur. J Arthroplasty 12(3):235-242

241. Tolk JJ, Waarsing JEH, Janssen RPA, van Steenbergen LN, Bierma-Zeinstra SMA, Reijman M (2019) Development of preoperative prediction models for pain and functional outcome after total knee arthroplasty using the dutch arthroplasty register data. J Arthroplasty 35(3):690-698

242. Tomek IM, Sabel AL, Froimson MI, Muschler G, Jevsevar DS, Koenig KM, et al (2012) A collaborative of leading health systems finds wide variations in total knee replacement delivery and takes steps to improve value. Health Aff (Millwood) 31(6):1329-1338

243. Tsai YS, Kung PT, Ku MC, Wang YH, Tsai WC (2018) Effects of pay for performance on risk incidence of infection and of revision after total knee arthroplasty in type 2 diabetic patients: A nationwide matched cohort study. PLoS One 13(11):e0206797

244. Varagunam M, Hutchings A, Black N (2015) Relationship between patient-reported outcomes of elective surgery and hospital and consultant volume. Med Care 53(4):310-316

245. Veroniki AA, Jackson D, Viechtbauer W, Bender R, Bowden J, Knapp G, et al (2016) Methods to estimate the between-study variance and its uncertainty in meta-analysis. Res Synth Methods 7(1):55-79

246. Vertullo CJ, Graves SE, Cuthbert AR, Lewis PL (2019) The effect of surgeon preference for selective patellar resurfacing on revision risk in total knee replacement: An instrumental variable analysis of 136,116 procedures from the australian orthopaedic association national joint replacement registry. J Bone Joint Surg Am 101(14):1261-1270

247. Voorn VMA, Marang-van de Mheen PJ, van der Hout A, So-Osman C, van den Akker-van Marle ME, Koopman-van Gemert A, et al (2017) Hospital variation in allogeneic transfusion and extended length of stay in primary elective hip and knee arthroplasty: A cross-sectional study. BMJ Open 7(7):e014143

248. Walldius B (1960) Arthroplasty of the knee using an endoprosthesis. 8 years' experience. Acta Orthop Scand 30:137-148

249. Warren J, Sundaram K, Anis H, Kamath AF, Mont MA, Higuera CA, et al (2019) Spinal anesthesia is associated with decreased complications after total knee and hip arthroplasty. J Am Acad Orthop Surg 28(5):e213-e221

250. Wei MH, Cheng CH, Li JY (2012) Discovering medical resource utilization in total knee arthroplasty (tka) using rule-based method. Arch Gerontol Geriatr 55(1):157-164

251. Wei MH, Lin YL, Shi HY, Chiu HC (2010) Effects of provider patient volume and comorbidity on clinical and economic outcomes for total knee arthroplasty: A population-based study. J Arthroplasty 25(6):906-912.e901

252. Welsh RL, Graham JE, Karmarkar AM, Leland NE, Baillargeon JG, Wild DL, et al (2017) Effects of postacute settings on readmission rates and reasons for readmission following total knee arthroplasty. JAMDA 18(4):367-e361

253. Wheeler BR (1984) Arthroscopic surgery of the knee: A review of early army experience. Mil Med 149(12):661-664

254. Wilson S, Marx RG, Pan TJ, Lyman S (2016) Meaningful thresholds for the volume-outcome relationship in total knee arthroplasty. J Bone Joint Surg Am 98(20):1683-1690

255. Wyatt MC, Hozack J, Frampton C, Hooper GJ (2019) Safety of single-anaesthetic versus staged bilateral primary total knee replacement: Experience from the new zealand national joint registry. ANZ J Surg 89(5):567-572

256. Yasunaga H, Tsuchiya K, Matsuyama Y, Ohe K (2009) Analysis of factors affecting operating time, postoperative complications, and length of stay for total knee arthroplasty: Nationwide web-based survey. J Orthop Sci 14(1):10-16

257. Yu TH, Chou YY, Tung YC (2019) Should we pay attention to surgeon or hospital volume in total knee arthroplasty? Evidence from a nationwide population-based study. PLoS One 14(5):12

258. Zhang W, Lyman S, Boutin-Foster C, Parks ML, Pan TJ, Lan A, et al (2017) Erratum to: Racial and ethnic disparities in utilization rate, hospital volume, and perioperative outcomes after total knee arthroplasty. J Bone Joint Surg Am 99(1):e30

259. Zhang W, Lyman S, Boutin-Foster C, Parks ML, Pan TJ, Lan A, et al (2016) Racial and ethnic disparities in utilization rate, hospital volume, and perioperative outcomes after total knee arthroplasty. J Bone Joint Surg Am 98(15):1243-1252

260. Zmistowski B, Restrepo C, Hess J, Adibi D, Cangoz S, Parvizi J (2013) Unplanned readmission after total joint arthroplasty: Rates, reasons, and risk factors. J Bone Joint Surg Am 95(20):1869-1876

1. Overall risk of bias was serious in all but one study in which it was moderate. [↑](#endnote-ref-2)
2. Same trend (consistent decrease with increasing hospital volumes) for studies accounting for >95% of patients; small studies with inconsistent effects. [↑](#endnote-ref-3)
3. p-Value of the largest study with ca. 80% of patients indicates significant effect. [↑](#endnote-ref-4)
4. Substantial unexplained heterogeneity beyond that expected by clinical differences in different health-care systems (*I*^2^ was >75% in main meta-analysis). [↑](#endnote-ref-5)
5. Overall risk of bias was moderate in 1 study (>80% of patients) and serious in two studies. [↑](#endnote-ref-6)
6. Confidence interval of the largest study (>80% of patients) or of the only study excludes no effect for the extreme comparison, but not for intermediate volumes. [↑](#endnote-ref-7)
7. Overall risk of bias was serious and moderate in 1 study each. Since the study with moderate risk of bias accounted for >85% of patients and events, we assume that the overall result is not very seriously biased. [↑](#endnote-ref-8)
8. Opposite direction of effect in the two studies. [↑](#endnote-ref-9)
9. Same trend was observed in two years investigated by the larger study (>95% of patients) [↑](#endnote-ref-10)
10. *p*-Value of the largest study with >60% of patients indicates significant effect. [↑](#endnote-ref-11)
11. Reason was the short duration of assessment (<3 months) in the study accounting for >99% of patients. [↑](#endnote-ref-12)
12. The direction of effect varied across studies [↑](#endnote-ref-13)
13. Confidence intervals of the largest studies are relatively narrow around no effect. [↑](#endnote-ref-14)
14. Overall risk of bias was serious. [↑](#endnote-ref-15)
15. Confidence intervals for highest and lowest volume category include no effect and results from a single were not replicated . [↑](#endnote-ref-16)
16. Overall risk of bias was serious in one study (62% of patients) and moderate in two studies. [↑](#endnote-ref-17)
17. Overall risk of bias was serious in all studies. [↑](#endnote-ref-18)
18. Confidence interval includes both a positive and a negative effect of increasing hospital and fails to exclude important benefits or hams. [↑](#endnote-ref-19)
19. Inconsistent effect across and within studies. [↑](#endnote-ref-20)
20. The largest study (70% of patients) only reports crude ratios. The only study with significant results includes only 7% of patients. [↑](#endnote-ref-21)
21. Overall risk of bias was moderate in all studies. [↑](#endnote-ref-22)
22. Single study, no replication of results from multiple studies available. [↑](#endnote-ref-23)
23. The direction of effect varied across years within the study. [↑](#endnote-ref-24)
